# Leptin receptor neurons in the dorsomedial hypothalamus input to the circadian feeding network

**DOI:** 10.1126/sciadv.adh9570

**Published:** 2023-08-25

**Authors:** Qijun Tang, Elizabeth Godschall, Charles D. Brennan, Qi Zhang, Ruei-Jen Abraham-Fan, Sydney P. Williams, Taha Buğra Güngül, Roberta Onoharigho, Aleyna Buyukaksakal, Ricardo Salinas, Isabelle R. Sajonia, Joey J. Olivieri, O. Yipkin Calhan, Christopher D. Deppmann, John N. Campbell, Brandon Podyma, Ali D. Güler

**Affiliations:** ^1^ Department of Biology, University of Virginia, Charlottesville, VA 22904, USA.; ^2^ Program in Fundamental Neuroscience, Charlottesville, VA 22904, USA.; ^3^ Department of Cell Biology, University of Virginia, Charlottesville, VA 22904, USA.; ^4^ Department of Biomedical Engineering, University of Virginia, Charlottesville, VA 22904, USA.; ^5^ Department of Neuroscience, School of Medicine, University of Virginia, Charlottesville, VA 22903, USA.; ^6^ Medical Scientist Training Program, School of Medicine, University of Virginia, Charlottesville, VA 22903, USA.

## Abstract

Salient cues, such as the rising sun or availability of food, entrain biological clocks for behavioral adaptation. The mechanisms underlying entrainment to food availability remain elusive. Using single-nucleus RNA sequencing during scheduled feeding, we identified a dorsomedial hypothalamus leptin receptor–expressing (DMH^LepR^) neuron population that up-regulates circadian entrainment genes and exhibits calcium activity before an anticipated meal. Exogenous leptin, silencing, or chemogenetic stimulation of DMH^LepR^ neurons disrupts the development of molecular and behavioral food entrainment. Repetitive DMH^LepR^ neuron activation leads to the partitioning of a secondary bout of circadian locomotor activity that is in phase with the stimulation and dependent on an intact suprachiasmatic nucleus (SCN). Last, we found a DMH^LepR^ neuron subpopulation that projects to the SCN with the capacity to influence the phase of the circadian clock. This direct DMH^LepR^-SCN connection is well situated to integrate the metabolic and circadian systems, facilitating mealtime anticipation.

## Teaser

Dorsomedial hypothalamus leptin receptor–expressing neurons are an information processing hub for meal timing.

## INTRODUCTION

When we eat is as important for our health as what and how much we eat. Studies in both mice and humans have shown that eating during the rest phase (daytime for mice or nighttime for humans) is associated with increased risk of weight gain, glucose intolerance, hepatic steatosis, and cardiovascular disease (*1*–*4*). Efforts to mitigate these deleterious effects in mice by restricting when they eat have provided remarkable promise to improve metabolic health and even extend lifespan. The potential benefits of time-restricted eating to human cardiometabolic health are the focus of many ongoing clinical studies (*5*–*9*). However, we have a limited mechanistic understanding of how meal timing influences our physiology and biological rhythms (*10*). Therefore, we sought to better understand the anatomical and molecular underpinnings of the interaction between feeding time and the circadian clock using a model of scheduled feeding (SF) that rapidly induces biological entrainment in rodents (*11*–*13*).

**Fig. 1.**
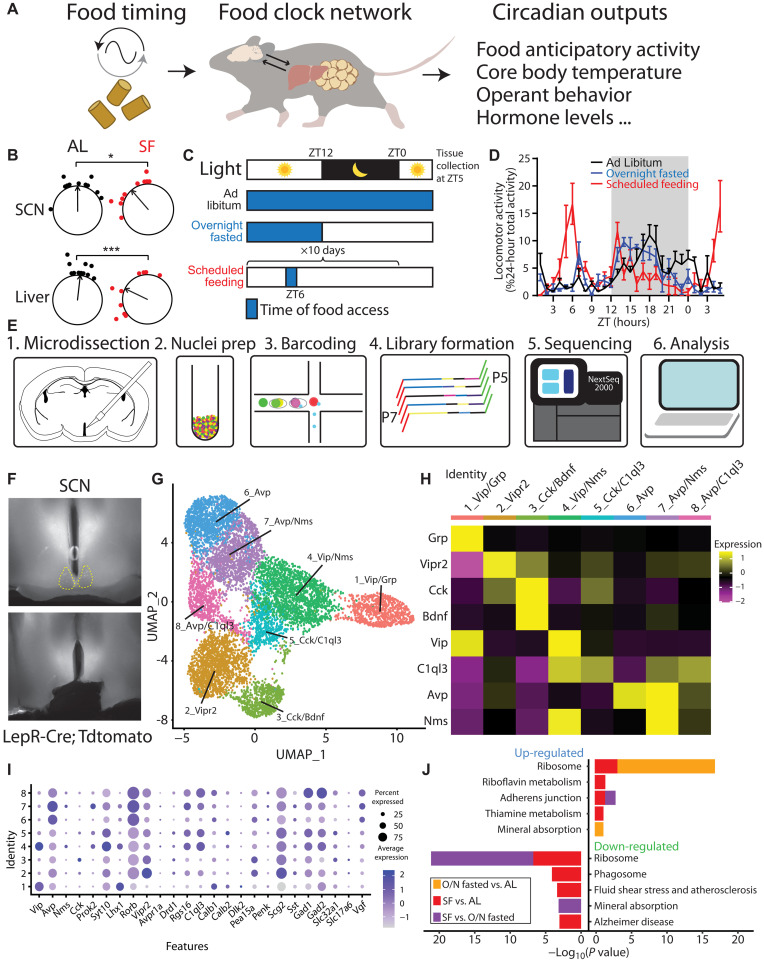
SCN snRNA-seq reveals minimal alteration of circadian genes during SF. (**A**) Diagram illustrating that food timing as a potent zeitgeber entraining an oscillatory network system in the brain and peripheral organs, relaying rhythmic behavior outputs. (**B**) The ZT phase of the first bioluminescence peak of SCN and liver from PER2::Luciferase (PER2LUC) mice that are either provided with scheduled food access for 4 days at ZT6 or ad libitum (AL) fed controls (untreated or given ZT6 saline injections). Phases of sample points are shown relative to the normalized mean phase of control SCN. Two-way analysis of variance (ANOVA) with Bonferroni post hoc comparison; *n* = 10 to 11 per group; *F*_treatment_(1,38) = 19.05, *P* < 0.001. (**C**) Schematic of experimental design. Mice were housed on a 12:12-hour light/dark (LD) cycle and either fed ad libitum, overnight (O/N) fasted, or provided a scheduled meal for 10 days at ZT6. Blue shading denotes food access. Mice were euthanized for tissue collection at ZT5. (**D**) Normalized locomotor activity starting 29 hours before tissue collection. *n* = 4 mice per condition. Data are represented as means ± SEM. (**E**) Schematic of single-nucleus RNA sequencing (snRNA-seq) workflow using 10× Genomics. (**F**) Representative images illustrating the area of dissection in the SCN for snRNA-seq. (**G**) Uniform Manifold Approximation and Projection (UMAP) plot of eight molecularly distinct SCN neuron subtypes (*n* = 8957 neurons). (**H**) Heatmap of cluster-average marker gene expression, scaled by gene. (**I**) Dot plot of average expression level (dot color) and percent expression (dot size) for each SCN neuron cluster. Genes shown are previously defined as SCN markers (*35*, *36*) and validated on the basis of Allen Brain Atlas Mouse Brain in situ hybridization data (*57*). (**J**) Gene enrichment analysis comparing top five pathways up- and down-regulated among feeding conditions in all SCN neurons, using KEGG Mouse 2019 database.

The suprachiasmatic nucleus (SCN) in the hypothalamus is the primary pacemaker that receives ambient light information from the retina, synchronizes circadian machinery throughout the body, and coordinates behavioral outputs (*14*, *15*). Less well understood, in the absence of a functional SCN (*12*, *13*), the circadian system retains the ability to entrain to the timing of nonphotic environmental cues, such as food (*11*). Numerous efforts have failed to identify any necessary genetic, molecular, or anatomic substrates of food entrainment (*16*, *17*). Emerging evidence suggests that the food entrainment system encompasses multiple food entrainable oscillators distributed across the central nervous system and peripheral organs, in which partial malfunction is compensated by other parts of the network (Fig. 1A) (*10*, *17*–*21*).

Here, we used a time- and calorie-restricted feeding paradigm to rapidly induce food entrainment in mice (*22*), with a focus on the SCN and the dorsomedial hypothalamus (DMH) that are involved in the regulation of feeding, locomotor activity, sleep-wake cycles, and hormone rhythms (*23*–*26*). By using single-nucleus RNA sequencing (snRNA-seq), we sought to identify neuronal populations in the SCN and DMH that show changes in circadian transcriptional programs. We did not observe appreciable transcriptional changes of genes associated with circadian rhythmicity or circadian entrainment pathways in the SCN during SF. However, we identified several neuronal populations in the DMH that altered their expression of circadian entrainment genes in response to timed feeding, including the leptin receptor (LepR)–expressing neurons. Next, we demonstrated that chronic silencing or overactivation of the DMH^LepR^ neurons, as well as leptin administration, impairs development of food entrainment. Last, we uncovered a direct neuronal projection from DMH^LepR^ neurons to the SCN and showed that DMH^LepR^ neuron stimulation is sufficient to phase shift the SCN circadian clock while altering the structure of circadian locomotor activity. These results define a mechanism that integrates mealtime information with the circadian clock via leptin signaling in the DMH.

## RESULTS

### SF alters circadian entrainment gene expression in the DMH but not the SCN

In mammals, the SCN in the hypothalamus is the seat of the primary circadian clock that receives ambient light signals and synchronizes the biological clocks distributed throughout the body (*14*). Although it is not required for the expression of food anticipatory behaviors (*13*, *27*, *28*), the SCN has recently been shown to modify the robustness of food entrainment [measured by one of the behavioral outputs of food entrainment, food anticipatory activity (FAA)] (Fig. 1A) (*29*). We tested the entrainment of central and peripheral circadian systems using an SF paradigm where we restricted both time and calories of the food delivered. In contrast to only time-restricted feeding regimens, which shift peripheral but not central circadian clocks (*30*), this SF paradigm induces a phase advance in the bioluminescent reported circadian rhythmicity of both the SCN and the liver from PER2::Luciferase (PER2LUC) transgenic mice (Fig. 1B and table S1) (*31*). Our observation is in line with previously demonstrated SCN rhythm phase shifts in time- and calorie-restricted animals (*32*, *33*). To further elucidate the transcriptional programs of hypothalamic regions in food entrainment, we harvested fresh brain tissues at 5 hours after lights on (zeitgeber time or ZT5) from mice that were subjected to three feeding conditions: ad libitum, overnight fasted, or fed at ZT6 for 10 days (SF; Fig. 1, C and D). We isolated SCN, as well as DMH, a hypothalamic region previously implicated in circadian and feeding regulation (Fig. 1E) (*23*, *34*). After brain region– and feeding condition–specific tissue collection and nucleus isolation, we performed snRNA-seq, yielding raw datasets of 59,708 and 65,837 cells from SCN- and DMH-containing tissues, respectively.

Using previously defined SCN markers (e.g., *Avp*, *Vip*, *Vipr2*, *Prok2*, and *Per2)* (*35*, *36*), we identified 8957 cells as SCN neurons and clustered them by transcriptomic similarity into eight candidate subtypes (Fig. 1, F to I, and fig. S1, A to D). In the final SCN dataset, the mean number of genes and unique transcripts [unique molecular identifiers (UMIs)] detected per cell in all SCN samples was 1783 and 3294, respectively (fig. S1C). We then compared SCN neuron gene expression across feeding conditions: SF versus ad libitum, SF versus fasting, and fasting versus ad libitum conditions. Using the Kyoto Encyclopedia of Genes and Genomes (KEGG) Mouse 2019 database, we performed gene enrichment analysis and identified the top five up- and down-regulated pathways in the SCN (Fig. 1J). Neither circadian entrainment nor circadian rhythm pathways were significantly altered in the SCN under SF relative to the other feeding conditions. Despite the primacy of the SCN in photically regulated pacemaking and the shift observed in *Per2* rhythmicity (Fig. 1B), these snRNA-seq data show that transcriptional alteration of the circadian system is limited in the SCN in response to food-based pacemaking, in line with previous work showing its expendability for food entrainment (*12*, *13*, *27*).

**Fig. 2.**
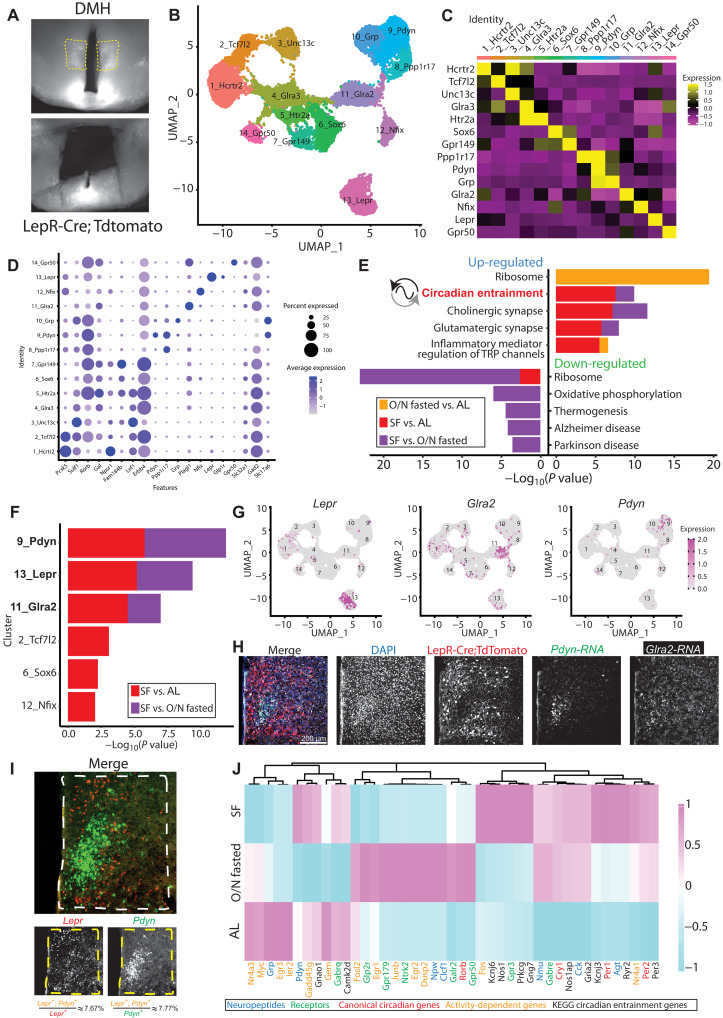
SF alters circadian entrainment genes in specific DMH neuron subtypes. (**A**) Representative images illustrating the DMH area dissected for snRNA-seq. (**B**) UMAP of 14 defined DMH neuron subtypes (*n* = 16,281 neurons). (**C**) Average gene expression heatmap labeled by cluster-specific markers in the DMH. (**D**) Dot plot of average expression level (dot color) and percent expression (dot size) of genes of interest within DMH clusters. These genes were either previously identified in DMH (*44*, *50*, *51*, *104*) or validated as DMH markers by the Allen Brain Atlas Mouse Brain in situ hybridization data (*57*). (**E**) Gene enrichment analysis comparing top five pathways up- and down-regulated across feeding conditions in all DMH neurons, using KEGG Mouse 2019 database. Inclusion criteria required *P* < 0.05 and log_2_ fold change > 0.25. (**F**) DMH clusters with differentially regulated circadian entrainment pathways in at least one SF comparison. (**G**) Feature plots indicating spatial expression of *Lepr* (left), *Glra2* (middle), and *Pdyn* (right), in DMH clusters. (**H**) Representative coronal section image localizing expression of LepR, *Pdyn*, and *Glra2* in the DMH. LepR cells were marked by LepR-Cre;TdTomato protein, whereas *Pdyn* and *Glra2* transcripts were visualized by RNA FISH. See also fig. S4F for zoomed-out view of the same brain section. (**I**) Representative RNA FISH coronal section image showing *Lepr* and *Pdyn* transcripts in the DMH. Quantification of *Lepr* and *Pdyn* coexpressing cells is depicted at the bottom. *n* = 3 mice. (**J**) Heatmap of select genes that were differentially expressed across feeding conditions in DMH^LepR^ neurons. TRP, transient receptor potential.

We next turned our attention to the DMH, a neighboring hypothalamic region that has been strongly implicated in circadian behaviors and physiological processes (*23*–*26*). However, the extent of DMH involvement in food entrainment is controversial with substantial reproducibility concerns (*17*, *18*, *26*, *37*–*49*), potentially due to the heterogeneity of DMH neurons. Therefore, we used snRNA-seq to compare the gene expression profiles of 16,281 DMH neurons from mice under ad libitum, fasted, or SF conditions. We first identified DMH neurons from our snRNA-seq dataset based on their enriched expression of known DMH markers including *Gpr50*, *Grp*, *Rorb*, *Sulf1*, *Pcsk5*, *Lepr*, *Pdyn*, and *Ppp1r17* (*44*, *50*, *51*). We then clustered these putative DMH neurons into 14 candidate subtypes according to transcriptomic similarity and annotated them based on top marker genes (Fig. 2, A to D, and fig. S1, E to H). The mean number of genes and UMIs per cell detected in all DMH samples was 2425 and 5235, respectively (fig. S1G). Our dataset contained clusters corresponding to previously identified DMH neuron populations, those expressing *Lepr*, *Pdyn*, or *Ppp1r17 (**42*–*44*, *51**)*, along with cluster-specific expression of 2_Tcf7l2 *(**52**)* or 12_Nfix that, together, were previously named *Lhx6*^+^ neurons (*53*). In sharp contrast to the SCN, DMH gene enrichment analysis revealed up-regulation of the circadian entrainment genes (e.g., *Kcnj6*, *Ryr2*, *Nos1*, etc.), which are involved in transmitting salient extracellular signaling cues to the core molecular clock (www.kegg.jp/entry/map04713; figs. S1I and S2) (*54*–*56*). This up-regulation was seen not only in SF versus ad libitum but also in SF versus fasted conditions, demonstrating that the effect on expression of circadian entrainment genes was not simply due to energy deficit but adaptation to food timing (Fig. 2E). These results imply that the genes capable of influencing the DMH circadian clock are altered by SF.

### SF alters circadian entrainment gene expression in DMH^LepR^ neurons

The DMH is a heterogeneous hypothalamic nucleus with multiple functions containing numerous genetically distinct cell populations, two of which (expressing either *Pdyn* or *Ppp1r17*) have been previously investigated in food entrainment behavior (*42*–*44*). Thus, we sought to understand that DMH neuronal subpopulations exhibit the most significant change in circadian entrainment gene expression during SF. Of the 14 neuron clusters we identified in the DMH, six showed differential gene expression in circadian entrainment pathway during energy deficit, and three of these had differential gene expression in both SF versus ad libitum and SF versus fasted conditions: cluster 9, Pdyn (prodynorphin) neurons; cluster 13, LepR neurons; and cluster 11, Glra2 (glycine receptor subunit alpha-2) neurons (Fig. 2, F to H, and figs. S3 and S4).

Of these candidate DMH neuron subtypes, those expressing *Pdyn* and *Lepr* have putative connections with both circadian and feeding regulation (*24*, *42*) and exhibit notably different anatomic distributions within the DMH, while *Glra2* does not (Fig. 2H and fig. S4, D to F) (*57*). For these reasons, we chose to further investigate the *Pdyn^+^* and *Lepr^+^* neuron subtypes in our dataset. *Lepr^+^* and *Pdyn^+^* neurons partially overlap in the DMH (*51*). However, using RNA fluorescence in situ hybridization (RNA FISH), we found that this overlap is minimal: only ~7.67% of *Lepr^+^* neurons are *Pdyn*^+^, while ~7.77% of *Pdyn^+^* neurons are *Lepr*^+^ (Fig. 2I). In addition, we observed that the *Lepr* expression is predominant in the dorsal and ventral DMH, whereas *Pdyn* expression is confined to the compact central DMH (Fig. 2I). The DMH^Pdyn^ neurons have been shown to entrain to SF (*42*) and dampen the robustness of FAA when silenced (*43*). However, the contribution of DMH^LepR^ neurons to food entrainment is unknown despite their important role in feeding and energy homeostasis (*24*, *51*, *58*, *59*). Therefore, we chose to focus our attention on DMH^LepR^ neurons. Detailed analysis of the DMH cluster 13 Lepr neurons in our dataset revealed that SF alters transcription of circadian entrainment pathway genes (figs. S1I and S4C), as well as activity-dependent genes, neuropeptides, receptors, and canonical circadian clock genes. Together, these transcriptional responses to SF point to a substantial role for DMH^LepR^ neurons in food entrainment (Fig. 2J and fig. S4C) (*42*, *43*).

### Leptin suppresses FAA and the calcium activity of DMH^LepR^ neurons

**Fig. 3.**
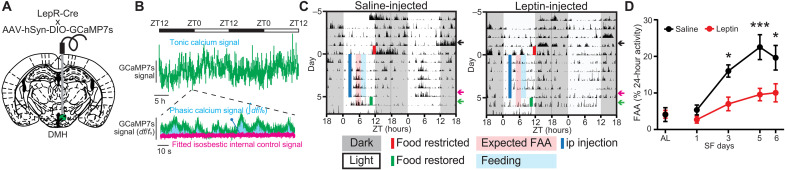
FAA during fiber photometry recordings is dampened by leptin. (**A**) Schematic diagram illustrating unilateral injection of AAV-hSyn-DIO-GCaMP7s and fiber optic cannula implantation to the DMH of LepR^Cre^ mice. (**B**) Example data trace illustrating two readouts of long-term fiber photometry calcium imaging. Tonic calcium signal represents the total fluorophore brightness. Phasic calcium signal represents the intracellular calcium activity over baseline activity in each recording session. See also fig. S5 for the second method of calculating these two readouts. (**C**) Representative locomotor actogram of single animals treated with saline (left) or leptin (right) during DMH^LepR^ neuron GCaMP7s fiber photometry recording. Mice are housed in 12:12-hour LD cycle, fasted at lights off on day 5 (solid red line), injected with saline or leptin at 2.5 hours after lights on (ZT2.5; solid blue line), and fed at ZT6 (2 g on days 1 and 2 and 2.5 g on remaining days). Red-shaded area is the FAA time window 2 hours before meal time (ZT4 to ZT6), and blue-shaded area is the first 2 hours after food delivery (ZT6 to ZT8). Food is restored at ZT10 on day 6 of SF. Color-coded arrows indicate 3 days that are selected for quantification in Fig. 4. ip, intraperitoneal. (**D**) Quantification of FAA during long-term DMH^LepR^ neuron GCaMP7s recording. FAA is defined as the locomotor activity in the 2-hour window before food delivery as a percentage of 24-hour activity. AL indicates ad libitum condition 2 days before initiation of drug administration. Mixed-effects (REML) analysis with Bonferroni post hoc comparison; *n* = 5 to 8 per group; *F*_treatment_(1,52) = 25.44, *P* < 0.001. Data are represented as means ± SEM. *P < 0.05; ***P < 0.001.

**Fig. 4.**
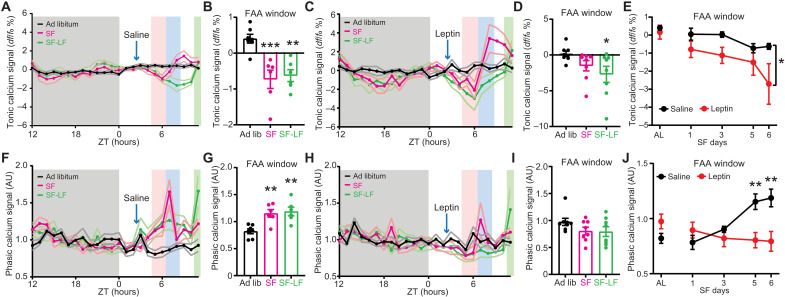
DMH^LepR^ neurons exhibit food entrainable calcium activity patterns which correlate with FAA. (**A**) Average tonic calcium signal of DMH^LepR^ neurons from saline control group 2 days before SF (black, ad libitum), 5th day during treatment (magenta, SF), and 6th day where saline injection was withheld and food was delayed for 3.5 hours (green, SF-LF). Shading color scheme is described in Fig. 3C. (**B**) Tonic calcium signal from saline treated mice in the FAA window (average of ZT5 to ZT6) from (A). *n* = 6 to 7 per group; *F*(2,16) = 12.27, *P* = 0.0006. (**C**) Average tonic calcium signal of DMH^LepR^ neurons from leptin-treated group. (**D**) Tonic calcium signal from leptin treated mice in the FAA window from (C). *n* = 8 per group; *F*(2,14) = 3.596, *P* = 0.0549. (**E**) Development of tonic calcium signal during FAA. *n* = 6 to 8 per group; *F*_treatment_(1,13) = 4.744, *P* = 0.0484. (**F**) Average phasic calcium signal of DMH^LepR^ neurons from saline control group. Normalized to average dark phase signal (ZT12 to ZT0). AU, arbitrary units. (**G**) Phasic calcium signal from saline-treated mice in the FAA window from (F). *n* = 6 to 7 per group; *F*(2,10) = 15.01, *P* = 0.0010. (**H**) Average phasic calcium signal of DMH^LepR^ neurons from leptin group. Normalized to average dark phase signal (ZT12 to ZT0). (**I**) Phasic calcium signal from leptin treated mice in the FAA window from (H). *n* = 8 per group; *F*(2,14) = 2.508, *P* = 0.1172. (**J**) Development of phasic calcium signal during FAA. *n* = 6 to 8 per group; *F*_treatment * time_(4,50) = 8.834, *P* < 0.0001. Data are represented as means ± SEM. *P < 0.05; **P < 0.01; ***P < 0.001. (B, E, G, and J) Mixed-effects (REML) analysis with Bonferroni post hoc comparison; (D and I) repeated-measures one-way ANOVA with Bonferroni post hoc comparison.

Since leptin is the ligand for LepR, we next sought to examine the effect of leptin on food entrainment. Predominantly made by adipose tissue, leptin is released in response to a meal (*60*), scaled to the time of day (*61*–*63*), and activates the Janus kinase/signal transducers and activators of transcription (STAT) pathway via phosphorylation of STAT3 in the DMH^LepR^ neurons (fig. S5, A and B) (*24*, *64*, *65*). We designed a paradigm to test whether dissociating the timing of leptin from food consumption is able to disrupt FAA, by administering leptin 3.5 hours in advance of SF. To simultaneously record intracellular calcium levels (as a proxy of neural activity) in the DMH^LepR^ neurons, we used an adeno-associated virus (AAV) to Cre-dependently express the calcium indicator GCaMP7s in the DMH of LepR-Cre mice (Fig. 3, A and B, and fig. S5, C and D) (*66*). When these animals were put on SF, we observed robust FAA development by day 3 in the saline control group, which was significantly suppressed by leptin administration (Fig. 3, C and D). Concurrently, DMH^LepR^ neurons rapidly increased their calcium signal at the time of food delivery, reproducing previous findings and confirming functionality of our system (fig. S5, E and F) (*51*). To evaluate the data on a circadian time scale, we extracted two readouts from the multiday fiber photometry calcium recordings (Fig. 3B): (i) the “tonic calcium signal” that is the overall intensity of the fluorescence normalized to a 24-hour moving average and (ii) the “phasic calcium signal” that is the acute calcium signal increases above the baseline during each recording session as a proxy of dynamic neuron bursts, normalized to the average of dark phase (Fig. 3B) (*66*–*68*). We analyzed both of these readouts using two separate methods that yielded similar results (Fig. 3B and fig. S5) (*60*–*62*). During SF, both types of calcium readouts from the DMH^LepR^ neurons developed responses that predicted the mealtime (Fig. 4 and fig. S5, G to S). Specifically, the overall fluorescence (tonic calcium signal) developed an anticipatory decrease before food access (Fig. 4, A and B, and fig. S5, I and J), which is in line with the documented anorexigenic role of DMH^LepR^ neurons (*51*, *58*, *59*). Unexpectedly, this dampened tonic calcium signal was not impaired by leptin injection but was slightly potentiated (Fig. 4, C to E, and fig. S5, K to M). In contrast, the phasic calcium signal increased during the FAA window in saline-injected control animals, which was absent in leptin-treated mice, in accordance with the suppressed development of FAA (Figs. 3, C and D, and 4, F to J; and fig. S5, F to H and N to S).

To ensure that the alteration of anticipatory DMH^LepR^ neuron calcium signal by leptin is due to defective food entrainment, rather than acute inhibition of neural activity, we withheld saline/leptin injections on day 6 and delayed food delivery for 3.5 hours [SF-late food (SF-LF)]. We observed that previously saline-treated mice still showed the anticipatory dampening of tonic and elevation of phasic calcium activity during the FAA window that remained until food delivery. In the previously leptin-treated group, DMH^LepR^ neurons did not exhibit an elevated phasic calcium signal even in the absence of exogenous leptin administration (Figs. 3, C and D, and 4; and fig. S5, G to S).

In summary, the baseline (tonic) neuronal activity of DMH^LepR^ neurons decreased in anticipation of scheduled food access, and this was further dampened by pre–meal leptin treatment. However, the anticipatory phasic calcium activity, which likely represents the acute increase in calcium transients (*24*, *51*, *58*, *59*), was largely abolished by pre–FAA leptin treatment. We therefore posit that the adaptation of DMH^LepR^ neuronal dynamics to SF time contributes to the development of the behavioral expression of food entrainment.

### Pre-FAA leptin suppresses development but not maintenance of FAA

**Fig. 5.**
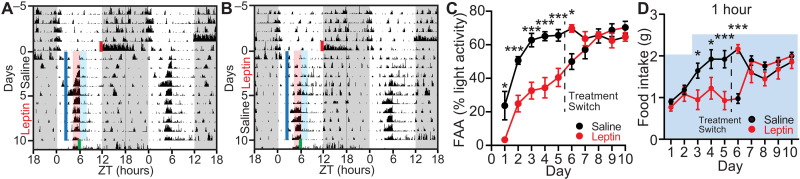
Pre-FAA leptin suppresses development but not maintenance of FAA. (**A** and **B**) Representative actograms of two mice on a 12:12-hour LD cycle under ad libitum conditions that are then subjected to SF beginning at lights off on SF day 0 and receiving either (A) saline (SF days 1 to 5) and then leptin (5 mg/kg; SF days 6 to 10) or (B) leptin (5 mg/kg; SF days 1 to 5) and then saline (SF days 6 to 10). Shading color scheme is described in Fig. 3C. See fig. S6 (A and B) for actograms of all animals. (**C**) Quantification of FAA. FAA is defined as the locomotor activity in the 2-hour window before food delivery as a percentage of light phase activity excluding the activity 1 hour after injection. Note that red (leptin) or black (saline) data markers indicate the treatment for the day, while the data connecting lines identify the initial leptin (red) or saline (black) treatment groups. Repeated-measures two-way ANOVA with Bonferroni post hoc comparison; *n* = 8 to 9 per group; *F*_treatment_(1,15) = 22.96, *P* < 0.001. (**D**) Food intake of mice during SF 1 hour after food delivery. Blue-shaded area indicates the total amount of food that was available for mice to consume on each day. Repeated-measures two-way ANOVA with Bonferroni post hoc comparison; *n* = 9 per group; *F*_time*treatment_(9,144) = 10.15, *P* < 0.0001. Data are represented as means ± SEM. *P < 0.05; ***P < 0.001.

To test to what extent leptin is able to suppress FAA, we designed a cross-over study of wild-type mice where we gave saline or leptin for the first 5 days of SF and switched the treatment group on the 6th day for five additional days of SF. As in previous experiments, we observed robust FAA by day 3 in the saline control group, while the development of FAA was significantly suppressed by leptin injections (Fig. 5, A to C, and fig. S6). On day 6, when the treatment was switched, the animals injected with leptin that previously received saline still exhibited robust FAA compared to those animals injected with saline but previously received leptin (Fig. 5, A to C). This demonstrates that the pre-FAA leptin is not merely masking entrainment but is instead impairing the establishment of the food timing machinery. Moreover, the FAA of the treatment groups was indistinguishable on day 7 (Fig. 5C), supporting the idea that the food entrainment system is a multinode network where disrupting one node slows its development but does not eliminate its establishment as the other parts of the system compensate.

Another observation we made during the cross-over experiment was that the delayed development of FAA correlated with a slower rate of food consumption (Fig. 5D). We sought to determine whether the rate of food intake, which defines the duration of food access, contributes to the speed of FAA development. To this end, we extended the duration of food access without changing the size of the meals by splitting the food into small pellets and delivering them in a prolonged time window (fig. S7). This paradigm still allowed for robust FAA development indicating that the duration of food access does not interfere with FAA development (fig. S7).

### Silencing DMH^LepR^ neurons impairs FAA

To determine whether DMH^LepR^ neurons are necessary for food entrainment, we chose to inhibit neuronal transmission by selectively expressing tetanus toxin (TeTx) in these neurons (fig. S8, A and B). As observed in DMH lesion studies or previous DMH^LepR^ neuron–silencing efforts, the behavioral circadian rhythmicity of animals was largely disrupted during ad libitum feeding under 12:12-hour light/dark (LD) cycle (fig. S8, C to J) (*23*, *24*, *39*). This implies a substantial role for DMH^LepR^ neurons in expression of circadian behaviors. Following SF, we observed impaired FAA in the TeTx-expressing animals compared to their mCherry controls even after 10 days of SF (fig. S8, C to J), indicating that the DMH^LepR^ neuronal output is essential for proper behavioral entrainment to SF.

### DMH^LepR^ neuron stimulation suppresses the development of FAA

**Fig. 6.**
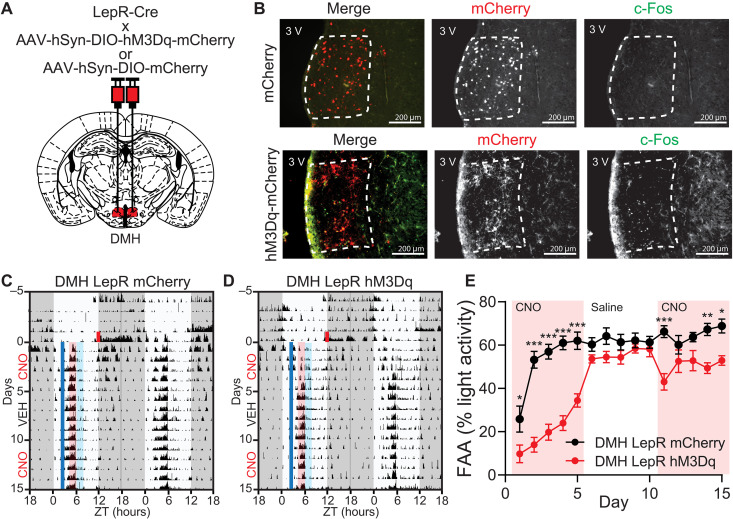
Pre-FAA activation of DMH^LepR^ neurons suppresses the development but not maintenance of food entrainment. (**A**) Schematic diagram illustrating bilateral injection of AAV-hSyn-DIO-hM3Dq-mCherry or AAV-hSyn-DIO-mCherry to the DMH of LepR Cre mice. (**B**) Representative images showing the expression of mCherry (top) or hM3Dq-mCherry (bottom) in DMH^LepR^ neurons and c-Fos response 2 hours after CNO injection. (**C** and **D**) Representative actograms of (C) DMH^LepR^ mCherry and (D) DMH^LepR^ hM3Dq mice on SF that received CNO (0.3 mg/kg; SF days 1 to 5), saline (SF days 6 to 10), and CNO (0.3 mg/kg; SF days 11 to 15) injection at ZT2.5. Shading color scheme is described in Fig. 3C. See fig. S9 (E and F) for actograms of all animals. (**E**) Quantification of FAA. Pink-shaded areas indicate days with CNO injection. No shading indicates saline injection. Repeated-measures two-way ANOVA with Bonferroni post hoc comparison; *n* = 8 to 9 per group; *F*_virus_ (1,15) = 46.24, *P* < 0.0001. Data are represented as means ± SEM. *P < 0.05; **P < 0.01; ***P < 0.001.

Although FAA is largely impaired when the DMH^LepR^ neurons are silenced by the selective expression of TeTx (fig. S8), these animals are also behaviorally arrhythmic during ad libitum conditions in the LD cycle (fig. S8I). To avoid this confound, we took an acute neuronal activity manipulation strategy using the DREADD hM3Dq to stimulate DMH^LepR^ neurons 3.5 hours before scheduled food time (Fig. 6, A and B). Similar to leptin treatment, we observed that premature activation of DMH^LepR^ neurons with clozapine N-oxide (CNO) injection impairs the development of FAA over the first 5 days of SF. However, it does not significantly impair the maintenance of FAA when CNO is administered after establishment of FAA (Fig. 6, C to E, and fig. S9).

These observations demonstrate that acute DMH^LepR^ neuron activation is not masking the behavioral outputs of food entrainment but instead is attenuating its development. This is in contrast to previous work where chemogenetic inhibition or activation of DMH^Ppp1r17^ neurons during SF showed no effect on FAA (*44*) and in agreement with our snRNA-seq findings that circadian entrainment pathway gene enrichment in DMH^Ppp1r17^ neurons is not altered during SF (Fig. 2). Notably, DMH^Pdyn^ neurons have been shown to entrain to SF (*42*) and to reduce the robustness of FAA when silenced (*43*). However, chemogenetic activation of DMH^Pdyn^ neurons only slightly suppressed FAA, which was mainly exhibited after the establishment of food entrainment (fig. S10). This underscores that DMH^LepR^ neurons are unique in their role during anticipation of a meal and their precisely timed activity is required for the development of FAA.

### DMH^LepR^ neuron stimulation alters landscape of circadian locomotor activity of ad libitum fed mice

**Fig. 7.**
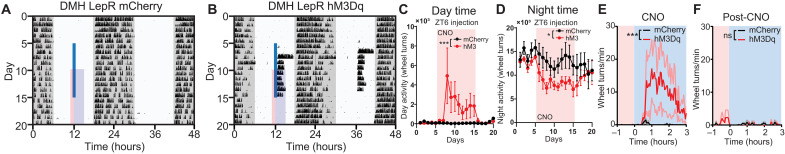
Light phase activation of DMH^LepR^ neurons induces locomotor activity. (**A** and **B**) Representative actograms of LepR Cre animals bilaterally injected with (A) AAV-hSyn-DIO-mCherry or (B) AAV-hSyn-DIO-hM3Dq-mCherry and injected with CNO (0.3 mg/kg) at ZT6 (solid blue line) in 12:12-hour LD cycle with access to a running wheel. Pink-shaded areas represent 1 hour before injection, and blue-shaded areas represent 3 hours after injection. See fig. S11 (A and B) for actograms of all animals. (**C**) Light phase (day) wheel revolutions of DMH^LepR^ mCherry and DMH^LepR^ hM3Dq animals before, during, and after CNO injections at ZT6 in 12:12-hour LD cycle. Pink-shaded areas represent days with CNO injection. Repeated-measures two-way ANOVA; *n* = 5 to 6 per group; *F*_virust * time_(19,171) = 3.302, *P* < 0.001. (**D**) Dark phase (night) wheel revolutions of DMH^LepR^ mCherry and DMH^LepR^ hM3Dq animals before, during, and after CNO injections at ZT6 in 12:12-hour LD cycle. Repeated-measures two-way ANOVA; *n* = 5 to 6 per group; *F*_virust * time_(19,171) = 1.707, *P* = 0.0390. (**E**) Average wheel running activity induced by chemogenetic activation of DMH^LepR^ neurons at ZT6 in 12:12-hour LD cycle. Color-coded time window is indicated in (A) and (B). Repeated-measures two-way ANOVA; *n* = 5 to 6 per group; *F*_virust * time_(79,711) = 2.519, *P* < 0.001. (**F**) Quantification of activity for 5 days after cessation of CNO injections. Repeated-measures two-way ANOVA; *n* = 5 to 6 per group; *F*_virus_(1,9) = 0.9519, *P* = 0.3547; *F*_virus * time_(79,711) = 1.202, *P* = 0.1215. Data are represented as means ± SEM. *P < 0.05; ***P < 0.001; ns, not significant.

**Fig. 8.**
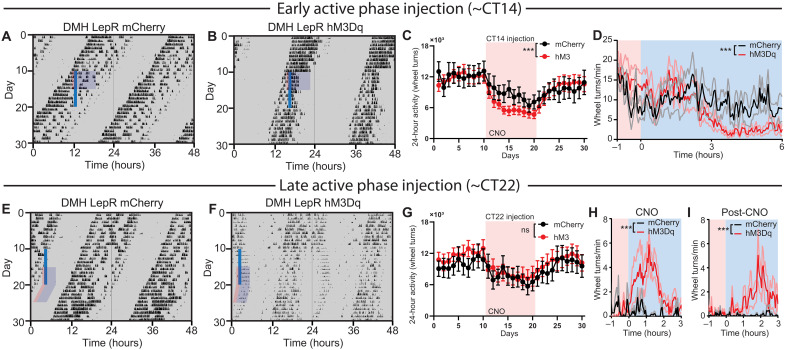
Repetitive activation of DMH^LepR^ neurons alters circadian locomotor activity in constant darkness. (**A** and **B**) Representative actograms of LepR Cre animals bilaterally injected with (A) AAV-hSyn-DIO-mCherry or (B) AAV-hSyn-DIO-hM3Dq-mCherry and injected with CNO (0.3 mg/kg) at ~CT14 (solid blue line), under ad libitum, constant dark conditions with access to a running wheel. See fig. S12 (A and B) for actograms of all animals. (**C**) Twenty-four–hour total wheel revolutions of before, during, and after CNO injections at ~CT14 in constant dark condition (DD). Repeated-measures two-way ANOVA; *n* = 9 to 12 per group; *F*_virust * time_(29,551) = 2.468, *P* < 0.001. (**D**) Average wheel running activity induced by chemogenetic activation of DMH^LepR^ neurons at ~CT14. Color coded time window is indicated in (A) and (B). Repeated-measures two-way ANOVA; *n* = 9 to 12 per group; *F*_virust * time_(140,2660) = 2.114, *P* < 0.001. (**E** and **F**) Representative actograms of LepR Cre animals bilaterally injected with (E) AAV-hSyn-DIO-mCherry or (F) AAV-hSyn-DIO-hM3Dq-mCherry and injected with CNO (0.3 mg/kg) at ~CT22 (solid blue line). See fig. S12 (C and D) for actograms of all animals. (**G**) Twenty-four–hour total wheel revolutions before, during, and after CNO injections at ~CT22 in DD. Repeated-measures two-way ANOVA; *n* = 9 to 12 per group; *F*_virus_ (1,19) = 0.2061, *P* = 0.6550; *F*_virus * time_(29,551) = 0.7120, *P* = 0.8676. (**H**) Average wheel running activity induced by chemogenetic activation of DMH^LepR^ neurons at ~CT22. Color-coded time window is indicated in (E-F). Repeated-measures two-way ANOVA; *n* = 8 to 12 per group; *F*_virus_(1,18) = 8.580, *P* = 0.0090; *F*_virus * time_(80,1440) = 2.029, *P* < 0.001. (**I**) Quantification of sustained activity for 5 days after cessation of CNO injections. Color-coded time window is indicated in (E) and (F). Repeated-measures two-way ANOVA; *n* = 8 to 12 per group; *F*_virus * time_(80,1440) = 1.745, *P* < 0.001. Data are represented as means ± SEM. ***P < 0.001; ns, not significant.

We next sought to characterize the impact of DMH^LepR^ neuron acute activation on general circadian behavior under energy-replete conditions. To this end, we stimulated the DMH^LepR^ neurons of ad libitum fed mice in the middle of the light phase of the 12:12-hour LD cycle, which acutely induced bouts of locomotor activity (Fig. 7, A to E, and fig. S11), in line with the previous literature (*69*). We observed a previously undescribed decrease in nighttime locomotor activity compared to controls (Fig. 7, A, B, and D, and fig. S11). Once injections were stopped after 10 days, daytime activity returned to normal levels immediately, although it took several days for nighttime activity to fully normalize (Fig. 7, C to F). To further parse the effects of DMH^LepR^ neuron stimulation on circadian locomotor activity, we repeated this experiment under ad libitum, constant dark conditions to remove the entraining effect of light. Notably, in constant darkness, DMH^LepR^ stimulation partitioned circadian locomotor activity without changing the free-running period (Fig. 8 and fig. S12). Activation early in the active phase [~circadian time (CT) 14] advanced the offset of locomotor activity to approximately 3 hours after CNO injection, with a concomitant reduction in 24-hour activity (Fig. 8, A to D, and fig. S12, A and B). Activation late in the active phase (~CT22) delayed the offset of locomotor activity to approximately 2 hours following CNO injection, with no change in total locomotor activity compared to controls (Fig. 8, E to I, and fig. S12, C and D). To our surprise, the delayed running wheel activity in DMH^LepR^ hM3Dq mice persisted for multiple days after cessation of CNO injections, in phase with the previous day’s injection time (Fig. 8, E to I). Notably, the clearance of CNO in the plasma is reported to be ~2 hours (*70*), while the DREADD-induced behavioral responses are exhibited up to ~10 hours (*71*)—both of which are much shorter than the observed time scale of behavior here (Fig. 8, E to I). These data demonstrate that the activation of DMH^LepR^ neurons can partition and entrain circadian locomotor activity in a time-dependent manner and suggest that DMH^LepR^ neurons are an integration hub that connects the food entrainable clock with the light entrainable clock.

### SCN is required for DMH^LepR^ neurons to alter circadian behaviors

**Fig. 9.**
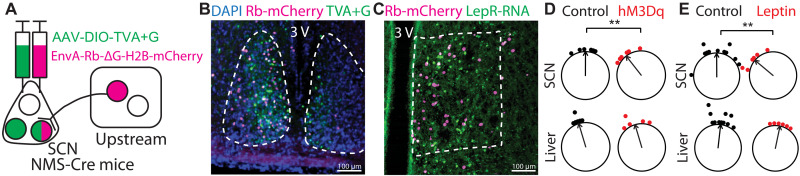
DMH^LepR^ neurons directly and functionally connect to the SCN. (**A**) Schematic illustration of NMS-Cre–dependent rabies virus monosynaptic retrograde tracing. (**B** and **C**) Representative images of (B) SCN and (C) DMH from retrograde tracing strategy in (A). (**D**) The ZT phase of the first bioluminescence peak of SCN and liver from PER2LUC mice injected with CNO at ZT6 (control) or PER2LUC;LepR-Cre mice with hM3Dq expressed in DMH^LepR^ neurons and injected with CNO at ZT6 (hM3Dq). Phases of sample points are shown relative to the normalized mean phase of control SCN. Two-way ANOVA with Bonferroni post hoc comparison; *n* = 5 to 6 per group; *F*_treatment_(1,19) = 5.361, *P* = 0.0319. (**E**) The ZT phase of the first bioluminescence peak of SCN and liver from control (mixture of ZT6 saline or untreated) or ZT6 leptin-injected PER2LUC mice. Phases of sample points are shown relative to the normalized mean phase of control SCN. Two-way ANOVA with Bonferroni post hoc comparison; *n* = 6 to 11 per group; *F*_treatment_(1,30) = 4.772, *P* = 0.0369. Control group is the same dataset as in Fig. 1B, replotted and analyzed with ZT6 leptin-treated animals. **P < 0.01.

While the DMH makes reciprocal connections with SCN neurons via direct and indirect routes, it is unknown whether DMH^LepR^ neurons project to the SCN (*72*). To identify direct DMH^LepR^ projections, we carried out Cre-dependent monosynaptic retrograde viral rabies tracing and found that DMH^LepR^ neurons innervate SCN neuromedin S (NMS) neurons (Fig. 9, A to C). To test the functionality of this connection, we selectively activated DMH^LepR^ neurons at ZT6 for 4 days using hM3Dq in PER2LUC mice and observed a phase advance in the SCN PER2 rhythm similar to animals on SF at ZT6. However, this stimulation did not induce a phase shift in the liver (Fig. 9D and table S1). Similarly, we observed that 4 days of leptin injection at ZT6 significantly phase advanced the SCN but not the liver (Fig. 9E and table S1). These data imply the existence of a leptin → DMH^LepR^ → SCN axis that communicates the metabolic status to the central circadian system.

**Fig. 10.**
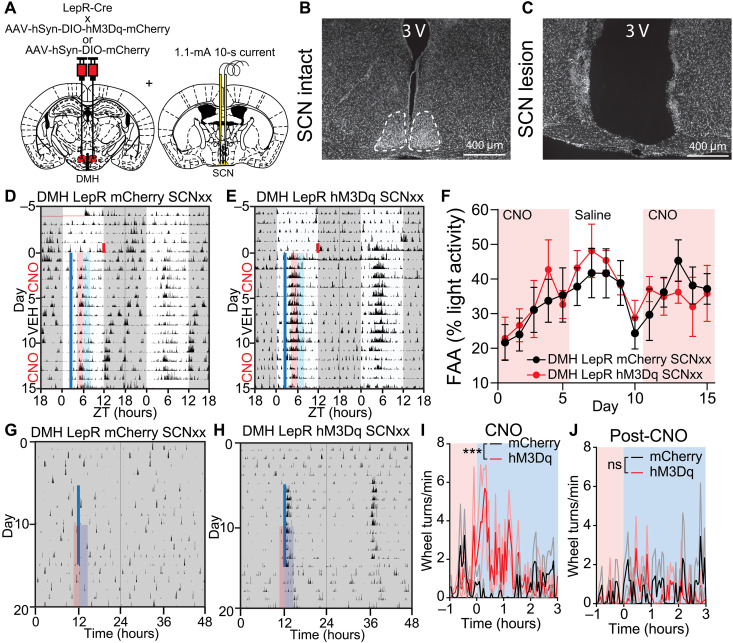
SCN is required for DMH^LepR^ neurons to alter circadian behaviors. (**A**) Schematic diagram illustrating bilateral injection of AAV-hSyn-DIO-hM3Dq-mCherry or AAV-hSyn-DIO-mCherry to the DMH of LepR-Cre mice coupled with electrolytic lesioning of the SCN in the same mice. (**B** and **C**) Representative 4′,6-diamidino-2-phenylindole (DAPI) staining images of (B) intact and (C) electrolytic lesioned SCN. (**D** and **E**) Representative actograms of SCN lesioned (D) DMH^LepR^ mCherry and (E) DMH^LepR^ hM3Dq mice on SF that received CNO (0.3 mg/kg; SF days 1 to 5), saline (SF days 6 to 10), and CNO (0.3 mg/kg; SF days 11 to 15) injection at ZT2.5. Shading color scheme is described in Fig. 3C. See fig. S13 (D and E) for actograms of all animals. (**F**) Quantification of FAA. Pink-shaded areas indicate days with CNO injection. No shading indicates saline injection. Repeated-measures two-way ANOVA with Bonferroni post hoc comparison; *n* = 6 to 7 per group; *F*_virus_(1,11) = 0.07566, *P* = 0.7884; *F*_virus * time_(14,154) = 0.4633, *P* = 0.9491. (**G** and **H**) Representative actograms of an SCN-lesioned (G) DMH^LepR^ mCherry and (H) DMH^LepR^ hM3Dq animal injected with CNO every 24 hours for 10 days in DD. Solid blue line indicates CNO injection. See fig. S13 (F and G) for actograms of all animals. (**I**) Average wheel running activity induced by chemogenetic activation of DMH^LepR^ neurons from (G) and (H). Color-coded time window is indicated in (G) and (H). Repeated-measures two-way ANOVA; *n* = 5 to 6 per group; *F*_virus_(1, 9) = 8.245, *P* = 0.0184; *F*_virust * time_(79,711) = 2.432, *P* < 0.0001. (**J**) Quantification of activity for 5 days after cessation of CNO injections from (G) and (H). Repeated-measures two-way ANOVA; *n* = 5 to 6 per group; *F*_virus_(1,9) = 0.3450, *P* = 0.5714; *F*_virus * time_(79,711) = 1.047, *P* = 0.3746. Data are represented as means ± SEM. ***P < 0.001; ns, not significant.

Last, we asked whether the SCN is necessary to mediate the effects of DMH^LepR^ neuron activation on circadian locomotor activity. We electrolytically lesioned the SCN bilaterally (SCNxx) in mice expressing hM3Dq or mCherry in the DMH^LepR^ neurons (Fig. 10, A to C) and subjected the mice to SF. Pre-FAA chemogenetic activation of DMH^LepR^ neurons in the absence of SCN no longer slowed down the development of FAA (Fig. 10, D to F, and fig. S13, A to E). When we chemogenetically activated DMH^LepR^ neurons at the same time of the day for 10 days in constant darkness with ad libitum food, we observed an acute bout of wheel running activity in SCNxx DMH^LepR^ hM3Dq, but not in SCNxx DMH^LepR^ mCherry control mice (Fig. 10, G to I, and fig. S13, F and G). In contrast to the SCN-intact experiments, we did not observe persistence of this activity in SCNxx animals following cessation of CNO injection on subsequent days (Fig. 10J and fig. S13, F and G). These results demonstrate that DMH activation–induced acute locomotor activity under ad libitum feeding condition is independent of the SCN. However, the SCN is necessary for DMH^LepR^ neurons to affect circadian behaviors during SF and ad libitum conditions.

## DISCUSSION

The circadian system synchronizes to salient nonphotic cues, such as timed availability of food, receptive mates, or exercise (*11*, *73*, *74*). In this work, we demonstrate that leptin, in combination with one of its central nervous system targets, the DMH^LepR^ neurons, forms an essential node that links food intake with the development of circadian food entrainment. In the process, we also identified an intriguing property of the circadian system whereby locomotor activity is partitioned into at least two components in response to overactivation of the DMH^LepR^ neurons. By functional direct innervation to the SCN, DMH^LepR^ neurons have the potential to serve as an information conversion point for nonphotic entrainment. The methodological paradigms presented here offer a platform to test the involvement of other molecular signals and anatomic regions in the development and maintenance of food entrainment and the relative function of inputs to the circadian system.

### Impact of SF on canonical clock genes in the SCN and DMH

In our snRNA-seq analysis of SCN and DMH, we did not observe a significant difference in the KEGG annotated circadian rhythm pathway (canonical circadian genes, e.g., *Bmal1* and *Clock*; www.kegg.jp/entry/map04710), except for *Per3* expression in the DMH (figs. S1I and S4C). However, we did observe a significant phase advance of PER2 bioluminescence in the SCN during SF (Fig. 1B). Therefore, we cannot rule out an alteration in the expression of other core circadian genes, since they are expressed at different phases, which might require snRNA-seq analysis at multiple time points across the day to reveal a change. In contrast, we did identify genes in the circadian entrainment pathway to be altered in the DMH (Fig. 2 and figs. S1I and S4C).

### Parameters of food entrainment

In addition to FAA, food entrainment is exhibited by other behaviors and physiological processes, sometimes with differential outcomes (Fig. 1A). For instance, peripheral organs, especially the liver, are more susceptible to food entrainment with the ketone body β-hydroxybutyrate as a key regulator (*21*, *30*, *75*). In addition, while the speed of entrainment to light is a widely accepted way to study the strength of photic entrainment (*76*–*78*), except for a few publications (*48*, *77*, *79*), food entrainment is exclusively gauged by existence of FAA or robustness of FAA at the maintenance stage. Our work highlights the importance of the development stage of food entrainment and places leptin and DMH^LepR^ neurons as a part of the food clock network.

### Leptin, DMH^LepR^ neurons, and their connections coordinate food entrainment

Previous findings regarding the role of leptin circadian regulation have been mixed. While leptin has been shown to advance the SCN when administered directly to brain slices of rats (*80*) or in vivo in mice (Fig. 9E), it does not appear to appreciably alter circadian behavior in mice (*81*). However, leptin suppresses FAA when administered continuously to leptin-deficient mice (*82*, *83*) or to rats in an activity-based anorexia model (*84*). The data presented here lend further credence to the importance of the timing of leptin. These findings highlight the need for future work investigating the role of mistimed leptin release as a result of snacking outside of mealtimes contributing to the adverse metabolic outcomes of modern eating habits (*85*).

It has been documented that the DMH serves as an anatomic integration hub to control food intake, thermogenesis, locomotor activity, and circadian behaviors (*24*, *51*, *65*, *69*, *86*). In addition to the DMH^LepR^-SCN connection described in this work, at least one other target of the DMH^LepR^ neurons, the agouti-related peptide (AgRP) neurons in the arcuate nucleus (ARC), has been implicated in the development of FAA (*87*, *88*). Therefore, we speculate that the DMH^LepR^ neurons, via at least their projections to the SCN and ARC^AgRP^ neurons, serve as a critical node for food entrainment by coordinating circadian and appetitive behaviors. This connectivity accommodates the previously demonstrated redundancy in the food entrainable network, as it has been convincingly shown that none of these individual regions are necessary for eventual development of food entrainment (*12*, *13*, *87*, *89*–*91*). However, ablation of one or more components does change the quality of food entrainment behaviors.

### Role of DMH^LepR^ neurons within the central pacemaker

Since the manipulation of DMH^LepR^ neurons does not ablate FAA once it is established, their role extends beyond merely masking the behavioral output of food entrainment (Fig. 6). Scheduled activation of DMH^LepR^ neurons under constant conditions induces a partitioned secondary bout of activity that remains even after cessation of the stimulation. This result supports the idea that DMH^LepR^ neurons play a role in providing input to the circadian pacemakers (Figs. 8 to 10). Therefore, we postulate the existence of at least two coupled but independent clocks within the DMH-SCN network akin to the “morning” and “evening” oscillators proposed in *Drosophila* (*92*). Recently, it was reported that dysfunction of SCN^CCK^ neurons can result in splitting of wheel running activity, likely due to the uncoupling of two clocks in the SCN (*93*). It remains to be determined whether the DMH and SCN are the two coupled clocks that can function independently in certain conditions or whether DMH partially uncouples the SCN which harbors these two clocks.

We observed that activation of DMH^LepR^ neurons in fed animals induces acute wheel running (Fig. 7B). Conversely, it inhibits FAA during SF (Fig. 6D). These contrasting observations suggest that a difference in energy status or food availability may be at play. This is similar to what has been observed following activation of AgRP neurons in the presence versus absence of food (*64*). However, the basis for energy state-dependent behaviors driven by DMH^LepR^ neurons has yet to be elucidated.

It has been increasingly acknowledged that when we eat, in addition to what and how much, plays a critical role in maintaining metabolic homeostasis and health (*4*–*6*, *8*, *94*–*96*). This bidirectional relationship between food and the circadian clock likely mediates dysfunction of both the circadian system and metabolic homeostasis in response to improperly timed feeding, e.g., nighttime snacking. Further understanding of the circuits that govern these food-circadian interactions will provide avenues to improve metabolic health (*9*, *97*–*99*).

## METHODS

### Single-nucleus RNA sequencing

#### Mouse lines

All experiments were carried out in compliance with the Association for Assessment of Laboratory Animal Care policies and approved by the University of Virginia Animal Care and Use Committee. Animals were housed on a 12:12-hour LD cycle with food (PicoLab Rodent Diet 5053) and water ad libitum unless otherwise indicated. For generation of the 10× snRNA-seq data (Figs. 1 and 2 and figs. S1 to S4), we used both male and female LepR-cre mice [B6.129(Cg)-Lepr^tm2(cre)Rck^/J, The Jackson Laboratory, #008320, RRID:IMSR_JAX:008320] (*100*) crossed to Ai14 tdTomato reporter line [B6.Cg-*Gt(ROSA)26Sor^tm14(CAG-tdTomato)Hze^*/J, strain #007914, RRID:IMSR_JAX:007914] (*101*).

#### SF in comprehensive lab animal monitoring system

Indirect calorimetry in the comprehensive lab animal monitoring system (CLAMS) system (Columbus Instruments) was used to evaluate metabolic parameters and ambulatory locomotor activity during ad libitum, overnight fasted, or time- and calorie-restricted SF. All mice were on a 12:12-hour LD cycle with ad libitum access to food and water unless otherwise indicated. LepR-Cre;tdTomato mice were singly housed and acclimated to the CLAMS for 3 days before experiment start. The night before SF began, the SF cohort was fasted, and cages were changed and thereafter were given 2 to 3 g of food, which is ~60% of the normal daily food intake (PicoLab Rodent Diet 5053) at ZT6 for 10 days. The overnight fasted cohort had ad libitum food access until the night before euthanasia, when food was removed and cages were changed. We repeated this experiment a total of three times with two mice per condition each time (*n* = 6 mice per condition total).

### Brain extraction and microdissection

All mice were euthanized at ZT5. Brains were immediately extracted and dropped into ice-cold Hanks’ balanced salt solution. After 2 min, brains were embedded in low melting point agarose (Precisionary Instruments, Natick, MA) and sectioned at 400 μm on a Compresstome VF-200 Vibrating Microtome (Precisionary Instruments, Natick, MA, USA) into deoxyribonuclease/ribonuclease-free 1× phosphate-buffered saline (PBS). Hypothalamic sections of interest were immediately collected into RNAprotect (QIAGEN, Hilden, Germany) and kept in RNAprotect at 4°C overnight. The next day Lepr-Cre;TdTomato–positive cells were visualized using a fluorescent stereoscope (Leica, Wetzlar, Germany). tdTomato fluorescence was used to approximate DMH and SCN boundaries during microdissection of these regions. DMH and SCN microdissected tissue samples were placed into Eppendorf tubes separated by brain region and feeding condition and stored in −80°C until nucleus isolation.

### Isolation of single-nucleus for RNA sequencing

DMH and SCN microdissected tissue samples were transferred from a -80°C freezer and into individual 2-ml glass dounce homogenizer tubes (Kimble, Vineland, NJ, USA) to be homogenized according to a protocol modified from the method described previously (*102*). Tissue was homogenized in 1 ml of buffer [16 mM sucrose, 5 mM CaCl, 3 mM Mg(Ac)_2_, 10 mM tris (pH 7.8), 0.1 mM EDTA, 1% NP-40, and 1 mM β-mercaptoethanol in H_2_O] on ice, with 25 passes of pestle A and 25 passes of pestle B. An additional 4 ml of buffer was added to the nucleus suspension and placed on ice for 5 min. Then, 5 ml of 50% OptiPrep density gradient medium (Sigma-Aldrich, MO, USA) [30 mM CaCl, 18 mM Mg(Ac)_2_, 60 mM tris (pH 7.8), 0.1 mM phenylmethylsulfonyl fluoride, and 6 mM β-mercaptoethanol in H_2_O; in 60% (w/v) solution of iodixanol in sterile water] was added to nuclei on ice and inverted 10 times to mix. The nucleus suspension was layered onto 10 ml of 29% OptiPrep solution (buffer + 50% OptiPrep) in a 38.5-ml Ultra-Clear tube (Beckman Coulter, CA, USA) before being centrifuged at 7333*g* for 30 min at 4°C. Supernatant was discarded, and the nucleus pellet was resuspended in 1× PBS + 1% bovine serum albumin (Sigma-Aldrich, MO, USA) + 2 mM Mg^2+^ + 0.1% ribonuclease inhibitor (Sigma-Aldrich, MO, USA) for 15 min on ice. The nucleus suspension was pipetted through a 20-μm mesh filter along with two drops of propidium iodide (PI) Ready Flow reagent (Thermo Fisher Scientific, MA, USA) and immediately taken on ice to be fluorescence-activated cell sorting sorted using an SH800 (Sony, Tokyo, Japan) cell sorter. Sorting was gated to select for PI^+^ single nucleus. Nuclei were sorted through a 70-μm nozzle into a 2-ml LoBind collection tube (VWR, PA, USA) containing 18.8 μl of RT Reagent B from the Chromium Next GEM (Gel Bead-In EMulsions) Single Cell 3′ Reagent Kit v3.1. The remaining components of the 10× Step 1 Master Mix were then gently mixed with the contents of the fluorescence-activated cell sorting collection tube and loaded into the 10× Genomics Chromium Controller Chip G.

### snRNA-seq workflow

The single-nucleus samples were processed into sequencing libraries using the Chromium Next GEM Single Cell 3’ Reagent Kit v3.1 according to the manufacturer’s protocol (version 3.1, revision D). After generation of the GEMs and reverse transcription of polyadenylated mRNA, cDNA was amplified (10 to 14 cycles), enzymatically fragmented, and ligated to Illumina adapters. Sequencing libraries were indexed, size-selected for 400 to 600 base pairs (bp) using SPRIselect (Beckman Coulter, Indianapolis, IN, USA), and quantified by Qubit (v4.0, 1× high-sensitivity dsDNA kit, Thermo Fisher Scientific) and Bioanalyzer (Agilent, Santa Clara, CA, USA). Size-corrected library concentrations were used to pool libraries for equimolar representation. The pooled concentration was measured by KAPA Library Quant quantitative polymerase chain reaction according to the manufacturer's instructions (KAPA Biosciences, Wilmington, MA) and by Qubit. Library pools were sequenced using a P100 cycle kit on the NextSeq 2000 (Illumina, CA, USA) in the University of Virginia School of Medicine Genome Analysis and Technology Core, RRID:SCR_018883. The sequencing structure was as follows: Read 1 was 28 bp (16 bp barcode, 12 bp UMI), read 2 was 98 bp (cDNA), and index 1 was 8 bp (single index). Overall, we had seven DMH and five SCN sequencing library pools. 10× batches 1 and 2 contained mixed pools of all feeding conditions, but 10× batch 3 contained a unique feeding condition per library pool (fig. S1, A and E).

### snRNA-seq data processing

Raw digital expression matrix files for each sequencing run were transferred to a high-performance cluster server where they were demultiplexed on the basis of sample index. We generated fastq files with bcl2fastq2 version 2.20.0 and then used Cell Ranger version 5.0.0 to align transcripts to the Cell Ranger supplied mouse genome, mm10 2020-A (GENCODE vM23/Ensembl 98), quantify expression levels, and partition them according to their cell-specific barcode. We ran the Cell Ranger count program with the “--include-introns” argument to include intronic reads in the gene expression quantitation.

### snRNA-seq analysis

Cell Ranger h5 files were read into Seurat v4 (*103*) in R (version 4.1.0) and RStudio (version 1.4.1717) and merged by brain region (DMH and SCN) for clustering analysis. We filtered the initial datasets to remove low-quality samples (i.e., cells with <100 genes detected or >0.5% mitochondrial reads). We then log-normalized the data, selected 2000 most variable genes (“feature selection”), and scaled gene expression. We performed principal components analysis to linearly reduce the dimensionality of the highly variable gene set. We defined distance metrics based on *K*-nearest neighbor analysis, grouped cells with Louvian algorithm modality optimization, and visualized cell embeddings in low-dimensional space with Uniform Manifold Approximation and Projection (UMAP) nonlinear dimensionality reduction. To focus our analysis on neurons, we subsetted neuronal clusters based on their enriched expression of neuronal marker genes (*Syt1*, *Syn1*, and *Tubb3*). To correct for batch effects, we integrated across sample batches using Seurat’s function for reciprocal principal components analysis. Next, we subsetted region-specific clusters based on expression of positive and negative marker genes for each target brain region (SCN and DMH), as described in the next section. We reclustered the identified DMH and SCN neurons using the following parameters: DMH, 2000 most variable genes, first 15 principal components, resolution setting of 0.8; SCN, 2000 most variable genes, first 13 principal components, resolution setting of 0.5 Last, we assessed cluster markers with the Wilcoxon rank sum test using Seurat default settings. Cluster markers were selected on the basis of top *P* values (adjusted to correct for multiple comparisons), high percent expression within the cluster, and low percent expression outside of the cluster and validated on the basis of Allen Brain Atlas mouse in situ hybridization data and previous literature.

### Identification of DMH and SCN neurons

DMH neuron types were selected on the basis of previously reported markers (*50*), as well as known highly expressed genes in the DMH including *Lepr*, *Pdyn*, *Ppp1r17*, *Cck*, and *Grp (**44*, *100*, *104**)*. Markers were validated via the Allen Brain Atlas mouse in situ hybridization data. In addition, clusters enriched with genes expressed in surrounding hypothalamic regions but not DMH were excluded from further analysis, including the following: paraventricular nucleus of hypothalamus (PVH) (*Sim1*) (*105*), ventromedial hypothalamus (VMH) (*Slit3*, *Qrfpr*, *Arpp21*, *Nr5a1*, and *Fezf1*) (*106*), ARC (*Prlr* and *Nr5a2*) (*107*), lateral hypothalamus (LH) (*Pvalb*, *Klk6*, and *Nts*) (*108*, *109*), or the tuberomammillary nucleus (*Hdc*) (*110*), (*111*). Cluster markers were prioritized on the basis of multiple comparison adjusted *P* values, high percent expression within the cluster, and low percent expression outside of the cluster. We defined SCN neuron populations based on previous literature by plotting expression of cluster specific markers and circadian genes from published datasets by our clusters (*35*, *36*, *72*).

### Functional analysis of differentially expression genes

To find differential gene expression between feeding condition groups (ad libitum, fasted, and SF), we used Seurat’s “FindMarkers” function to run Wilcoxon rank sum statistical tests. False discovery rate (FDR) was calculated using the “p.adjust” function. We set cutoffs of log_2_ fold change > 0.25 and FDR < 0.05 to quantify the total number of differentially expressed (DE) genes per feeding condition comparison. To visualize STRING functional protein interaction networks, we used Cytoscape open-source software (version 3.9.1). We input lists of DE genes in the cluster 13_Lepr between both SF and fasted and SF and ad libitum using the same criteria as previously described above. We ran KEGG functional enrichment analysis on the mouse genome to label genes involved in up-regulated pathways during SF. We measured DE pathways among the three feeding conditions using the “enrichR” package. To visualize genes and pathways differing significantly between feeding conditions, we used the “DEenrichR” function that applies the Wilcoxon rank sum test to identify DE genes [log_2_(fold change) > 0.25 and multiple comparison adjusted *P* < 0.05). Significantly DE genes are then scored on the basis of odds ratios to fall into pathways categories defined by the KEGG Mouse 2019 database. With the “ggplot2” package, we then graphed up-regulated and down-regulated pathways in each comparison on a superimposed bar graph, colored by condition-specific comparison, and ranked by maximum −log_10_(*P* value).

### RNA fluorescence in situ hybridization

RNA FISH was performed on fixed brain slices with a probe to detect LepR and Pdyn RNA (RNAscope Multiplex Fluorescent Reagent Kit v2 Assay, Advanced Cell Diagnostics [ACD]). All procedures were carried out according to the manufacturer's instructions. Briefly, sections were pretreated with RNAscope hydrogen peroxide to block the activity of endogenous peroxidases. After a wash in distilled water, sections were permeabilized with RNAscope protease IV for 30 min at 40°C. Sections were hybridized with the Lepr (ACD, catalog no. 402731), Pdyn (ACD, catalog no. 318771), and Glra2 (ACD, catalog no. 510301) probe at 40°C for 2 hours, followed by amplification incubation steps: Amp 1, 30 min at 40°C; Amp 2, 30 min at 40°C; Amp 3, 15 min at 40°C. Horseradish peroxidase (HRP) signals were developed with RNAscope Multiplex FL v2 HRP and TSA Plus fluorophores (HRP-C1 and 1:750 TSA Plus Cy3 for Lepr, Cy2 or Cy5 for Pdyn, and Cy5 for Glra2). Sections were washed with the provided washing buffer 2 × 2 min in between each step. Sections were then coverslipped with 4in between each step. Sectio (DAPI) Fluoromount-G (Southern Biotech). Confocal microscope imaging was performed on a Zeiss LSM 800 microscope (Carl Zeiss).

### Automated quantification for RNA FISH images

RNA FISH–labeled cells were counted using CellProfiler image analysis software, with an analysis pipeline modified from previously published work (*112*). Briefly, DAPI staining of nuclei was used to identify cells, and then cells with more than three stained speckles, or >60% of cell area covered by staining, were considered as positive for the marker.

### Behavioral assays

#### Mice

All experiments were carried out in compliance with the Association for Assessment of Laboratory Animal Care policies and approved by the University of Virginia Animal Care and Use Committee (Figs. 3 to 10 and figs. S5 to S13). Animals were housed on a 12:12-hour LD cycle with food (PicoLab Rodent Diet 5053) and water ad libitum unless otherwise indicated. All experiments were performed on male mice 12 weeks or older unless otherwise indicated. Wild-type C57BL6/J mice, LepR-Cre [B6.129-Lepr^tm2(cre)Rck^/J, The Jackson Laboratory, #008320, RRID:IMSR_JAX:008320] (*100*), Ai14 tdTomato reporter line [B6.Cg-*Gt(ROSA)26Sor^tm14(CAG-tdTomato)Hze^*/J, strain #007914, RRID:IMSR_JAX:007914] (*101*), and Pdyn-Cre [B6;129S-Pdyn^tm1.1(cre)Mjkr^/LowlJ, The Jackson Laboratory, #027958, RRID:IMSR_JAX:027958] (*113*) mice were used.

### Scheduled feeding

For SF, mice were first acclimated to single housing for 7 day4s, followed by acclimation to infrared (IR) beam interruption chambers [Columbus Instruments, or custom built (*114*)] for a minimum of 72 hours before starting the recording of locomotor activity. After at least 3 days of recording of baseline locomotor activity while the mice had ad libitum access to food, mice were fasted at lights off (ZT12) on day 0 of SF, along with a full cage change. Mice were weighed, and injected with vehicle (saline), leptin (5 mg/kg), or CNO (0.3 mg/kg) at ZT2.5. Mice were then refed 3.5 hours later at ZT6 (ZT6.5 for fiber photometry experiment in Fig. 3). During the first 2 days, mice were fed 2 g, after which they were fed 2.5 g, which is ~60% of the normal daily food intake (PicoLab Rodent Diet 5053). For the experiment in which we extended the food delivery window (fig. S7), the same amount of food (2 g on first 2 days and 2.5 g on days 3 to 10) was given to both control and extended groups. In the extended group, food was evenly split to four pellets and delivered at ZT6, ZT7, ZT8, and ZT9. Control group received the whole pellet of food at ZT6 as other SF experiments in this work. In the leptin treatment experiments (Fig. 5 and fig. S6), after 5 days, treatment groups were switched, so that mice previously given saline received leptin and vice versa for the remaining 5 days. In DREADD experiments (Figs. 6 and 10 and figs. S9, S10, and S13), CNO was administered for 5 days, followed by 5 days of saline before switching back to 5 days of CNO administration. FAA was quantified as the amount of locomotor activity expressed in the 2-hour window before food delivery (ZT4 to ZT6), and normalized to 24-hour activity (Figs. 1 and 3) or total light phase locomotor activity (in all other figures). In case of injection at ZT2.5, the 1 hour of activity after injection was excluded to eliminate handling induced locomotion. Wild-type groups injected with saline or leptin were age- and weight-matched. Surgery operated groups were age-matched.

### Stereotactic surgery

Animals were anesthetized with isoflurane (5% induction and 2 to 2.5% maintenance; Isothesia) and placed in a stereotaxic apparatus (Kopf). A heating pad was used for the duration of the surgery to maintain body temperature, and ocular lubricant was applied to the eyes to prevent desiccation. A total of 500 nl of AAV [AAV8-hSyn-DIO-hM3Dq-mCherry, plasmid from Addgene, #44361, RRID:Addgene_44361 (*115*), virus packed at UNC Vector Core; AAV8-hSyn-DIO-mCherry plasmid from Addgene #50459, RRID:Addgene_50459 (*115*), and virus packed at UNC Vector Core; AAV1-hSyn-DIO-GCaMP7s virus from Addgene #104491-AAV1, RRID:Addgene_104491 (*116*); AAV1-CBA-DIO-GFP-TeTx (*117*) was generously gifted by L. Zweifel (University of Washington, Seattle, WA)] was delivered using a 10-μl syringe (Hamilton) and 26-gauge needle (Hamilton) at a flow rate of 100 nl/min driven by a microsyringe pump controller (World Precision Instruments, model Micro 4). The syringe needle was left in place for 10 min and was completely withdrawn 17 min after viral delivery. For in vivo calcium imaging, an optic fiber guide cannula was implanted unilaterally following viral delivery, at 0.2-mm dorsal to the viral injection coordinates, and stabilized on the skull with dental cement (C&B METABOND, Parkell). For electrolytic lesions, a parylene-insulated, tip-exposed 2-megohm tungsten electrode was placed bilaterally into the SCN, and a current of 1.1 mA was applied for 11 s. Two weeks minimum were allowed for recovery and transgene expression after surgery. Stereotaxic coordinates relative to Bregma (George Paxinos and Keith B. J. Franklin): SCN, mediolateral (ML): ±0.3 mm, anterior posterior (AP): −0.35 mm, dorsoventral (DV): −5.75 mm; DMH, ML: ±0.3 mm, AP: −1.8 mm, DV: −5.45 mm. After the surgery, the animals were housed individually. All surgical procedures were performed under sterile conditions and in accordance with University of Virginia Institutional Animal Care and Use Committee guidelines. Histological analysis was performed to validate the success of intracranial surgeries. Animals with unsuccessful viral/lesion/implant targeting were excluded.

### Histological analysis and imaging

For fixed tissue collection, animals were deeply anesthetized (ketamine:xylazine, 280:80 mg/kg, intraperitoneally) and perfused intracardially with ice-cold 0.01 M phosphate buffer solution (PBS), followed by fixative solution [4% paraformaldehyde (PFA) in PBS at a pH of 7.4]. For testing the functionality of hM3Dq (Fig. 6B), CNO (0.3 mg/kg) was intraperitoneal injected at 2 hours before perfusion and brain harvesting. After perfusion, brains were harvested and postfixed overnight at 4°C in PFA. Fixed brains were then transferred into 30% sucrose in PBS for 24 hours and then frozen on dry ice. Frozen brains were sectioned immediately or stored in −80°C for future processing. Coronal sections (30 μm) were collected with a cryostat (Microm HM 505 E). Sections were permeabilized with 0.3% Triton X-100 in PBS (PBS-T) and blocked with 3% normal donkey serum (Jackson ImmunoResearch, RRID:AB_2337258) in PBS-T (PBS-T DS) for 30 min at room temperature. Sections were then incubated overnight at 4°C (or otherwise indicated) in primary antibodies diluted in PBS-T DS. For visualization, sections were washed with PBS-T and incubated with appropriate secondary antibodies diluted in the blocking solution for 2 hours at room temperature. Sections were washed three times with PBS and mounted using DAPI Fluoromount-G (Southern Biotech). Images were captured on a Zeiss Axioplan 2 Imaging microscope equipped with an AxioCam MRm camera using AxioVision 4.6 software (Zeiss). The following primary antibodies was used for fluorescent labeling: anti–c-Fos (rabbit, 1:1000; Synaptic Systems, #226003, RRID:AB_2231974), anti-DsRed (rabbit, 1:1000; Takara Bio, catalog no. 632496, RRID:AB_10013483), anti-TdTomato (goat, 1:1000; Arigobio, catalog no. ARG55724), and anti-pSTAT3 (Tyr705)(D3A7) (rabbit, 1:250; Cell Signaling Technology, catalog no. 9145, RRID:AB_2491009). The secondary antibodies (Jackson ImmunoResearch) used was Cy2-conjugated donkey anti-rabbit (1:250; catalog no. 711-225-152, RRID:AB_2340612), Cy3-conjugated donkey anti-rabbit (1:250; catalog no. 711-165-152, RRID:AB_2307443), and Cy3-conjugated donkey anti-goat (1:250; catalog no. 705-165-147, RRID:AB_2307351).

### Antigen retrieval for pSTAT3 staining

Antigen retrieval was performed before immunohistochemistry staining of pSTAT3, by incubating the sections in the following solutions sequentially in room temperature: 1% NaOH + 0.3% H_2_O_2_ in PBS for 20 min, 0.3% glycine in PBS for 10 min, and 0.03% SDS in PBS for 10 min. Then, antigen retrieval–treated sections were stained following the immunohistochemistry staining procedures described.

### Retrograde tracing

In rabies virus tracing, 200 nl of AAV1-synP-FLEX-splitTVA-EGFP-B19G (Addgene, #52473-AAV1) was injected to SCN of NMS-Cre mice using the stereotactic surgery method described above. Three weeks after AAV injection, 120 nl of EnvA-dG-Rabies-H2B-mCherry (Salk Viral Vector Core) was delivered to the same coordinates. After one more week, fresh brains were harvested and processed for antibody labeling or RNA FISH probing as described above.

### Bioluminescence

To determine the treatment effect on the phase of molecular circadian rhythm of the liver or SCN, PER2LUC, or PER2LUC;LepR-Cre mice were individually housed under 12:12-hour LD cycle for four consecutive days and received one of the following treatments: (i) leptin (5 mg/kg) at ZT6, (ii) CNO (0.3 mg/kg) at ZT6, (iii) SF feeding at ZT6, and (iv) saline at ZT6 or no treatment, pooled as control (used for both Figs. 1B and 9E). On the 5th day, mice were euthanized between ZT5 and ZT10. Brains were immediately extracted and dropped into ice-cold Hanks’ balanced salt solution. After 2 min, brains were embedded in low melting point agarose (Precisionary Instruments, Natick, MA) and sectioned at 300 μm on a Compresstome VF-200 Vibrating Microtome (Precisionary Instruments, Natick, MA, USA). Brain tissue containing the SCN and liver tissue were dissected for bioluminescence recording using a method adapted from previous work (*118*). SCN slices and liver tissues were cultured in 35-mm culture dishes with 1.2 ml of Dulbecco’s modified Eagle’s medium (Sigma-Aldrich, D5030) supplemented with d-glucose (3.5 g/liter), 2 mM GlutaMAX (Gibco), 10 mM Hepes, penicillin/streptomycin (25 U/ml), 2% B-27 Plus (Gibco), and 0.1 mM d-luciferin sodium salt (Tocris). The culture dishes were covered with 40-mm-diameter glass cover slides, sealed by high-vacuum grease (Dow Corning), and maintained in a nonhumidified incubator at 36.8°C. Bioluminescence from firefly luciferase in each of SCN slices or liver sections was recorded in 10-min intervals by a 32-channel/4-photomultiplier tube luminometer LumiCycle (Actimetrics) in the incubator. The bioluminescence data were collected and analyzed by LumiCycle Analysis software (Actimetrics). The time of the first peak in bioluminescence was manually determined from each tissue sample. Each data point represents the average of two tissue samples from the same animal that was then used for further statistical analysis.

### In vivo fiber photometry recording

The viral vector and fiber optic implant were delivered to the target brain area within one surgical procedure as described above. Experiments were initiated at least 2 weeks after surgery to allow for recovery and transgene expression. For long-term recordings, mice were individually housed in their home cages and transferred to the recording room 3 days before experiment to acclimate to the environment. Implanted fiber-optic cannula (Thorlabs, 0.39 numerical aperture, Ø200-μm Core) was connected to fiber-optic cable (Doric Lenses, 0.37 numerical aperture, Ø200-μm Core) with the use of a zirconia mating sleeve (Doric Lenses). The fiber-optic cables were wrapped with metal sheath (Doric Lenses) to prevent breakage and connected to rotary joints (Doric Lenses) to allow free movement. The fiber photometry system used in this work records the fluorescent signal from both calcium-dependent (465 nm) and calcium-independent isosbestic (405 nm) excitation light wavelengths, in which the isosbestic wavelength excitation signal is used to control for the artifacts from animal movement, fluorescent reporter expression, and photobleaching. The signal was collected at 120-Hz sampling rate. To limit the photobleaching of the fluorescent reporter during the long-term recording, the signal was only collected for 2 min every 12 min (for three of seven saline-treated mice and one of eight leptin-treated mice in the SF experiment, the data were collected for 10 min per hour. These datasets were included in analysis when possible. See below for details). Animals that did not exhibit Ca^2+^ signal increase in response to food presentation were considered mistargeted and were excluded from the analysis (fig. S5, E to H).

### Fiber photometry data analysis

For food response during the SF experiment, the food was delivered 1 min after the initiation of the 2-min recording session. The variability caused by signal strength was normalized by calculating *z* score [(signal-average of baseline signal) / SD of baseline signal] for food response experiments where the first 15 s of the 2-min sessions was used as baseline. When processing long-term recording results for circadian analysis, data were processed in MATLAB using a combination of methods modified from work described previously (*66*, *67*, *93*). To calculate the tonic calcium signal, the calcium dependent (465-nm excitation) signal was averaged by hour and divided by hourly average of calcium-independent isosbestic (405-nm excitation) signal, to get artifact-corrected fluorescent signal at time *t*, *F*(*t*). The fluorescence change over baseline fluorescence (Δ*F/F*_0_) was further calculated as [*F*(*t*) *− F*_0_) / *F*_0_, in which *F*_0_ was the 24-hour moving average surrounding time *t* (Fig. 4). Results were also calculated as *z* score: [*F*(*t*) *− F*_0_) / STD_0_, in which *F*_0_ and STD_0_ were the 24-hour moving average and SD surrounding time *t* (fig. S5).

To calculate the phasic calcium signal and eliminate the artifact from motion or protein expression, the calcium-independent isosbestic (405-nm excitation) signal was fitted to the calcium-dependent (465-nm excitation) signal with a linear least-squares fit. The fluorescence change over baseline fluorescence (Δ*F/F*_0_) was calculated as (465-nm induced signal − fitted 405-nm induced signal) / (median of 465-nm induced signal). Notably, in the long-term experiments where multiple short-term sessions were recorded, *F*_0_ was the median for the entire experiment for consistency. Further, in each recording session, the fitted 405-nm induced signal was defined as the estimated cutoff line, so that the values on Δ*F/F*_0_ curves below 0 were cutoff. The phasic calcium signal is the integral of Δ*F/F*_0_-adjusted curve (Fig. 3B) and represents the intracellular calcium activity during that session (Figs. 3 and 4). The calculated phasic calcium signal was further averaged by hour and normalized to the average of the 12-hour dark phase signal in each given day. To account for the concern that 465- and 405-nm induced signals have different rates of photobleaching, a second method was used to calculate phasic calcium signal (fig. S5). In this method, the fluorescence change over baseline fluorescence (Δ*F/F*_0_) was calculated as (465-nm induced signal − fitted 405-nm induced signal) / (30-s sliding 10th percentile of 465-nm induced signal). Next, 10th percentile of Δ*F/F*_0_ signal in a 30-s sliding window (*119*) was used as the cutoff line to calculate phasic calcium signal.

### Circadian behavioral analysis

Locomotor activity data were collected by wheel-equipped cages (Nalgene) or IR beam interruption chambers [Columbus Instruments, or custom built (*114*)] in light-tight compartments under a 12:12-hour LD cycle. Fluorescent lights (~400 lux) were used for behavioral experiments. Food and water were provided ad libitum unless otherwise indicated. Wheel running activity was monitored and analyzed with the ClockLab collection and analysis system (Actimetrics, Wilmette, IL). IR beam interruption activity data were analyzed in Excel for quantification or converted to a ClockLab supported file format for circadian analysis.

### Statistical analysis and reproducibility

To compare the effects of treatment over time, two-way analysis of variance (ANOVA) test was used. In experiments with a single variable and more than two groups, one-way ANOVA was performed. In case data points were missing because of technical failure (i.e., power outage during long-term recording), where repeated-measures ANOVA was not possible to perform, mixed-effects [Restricted maximum likelihood (REML)] analysis was used instead. Following a significant effect in the ANOVA test, Bonferroni post hoc comparison was used to determine differences between individual data points. Analyses were conducted using GraphPad Prism 8 statistical software for Windows. All data were presented as means ± SEM with *P* < 0.05 considered statistically significant.

Power analysis was not done specifically for each experiment in this work to determine the sample size. However, Cohen's *D* analysis determined that for *a* = 0.05 and power = 0.8, ~6 samples are enough to identify ~40% difference in mean with ~25% SD and ~12 samples are enough to identify ~20% difference in mean with ~20% SD. Therefore, the sample size in this work ranges from 6 to 12 for each group. The “*n*” numbers for each experiment indicated in figure legends are biological replicates (number of animals). Littermates were randomly assigned to control or treatment groups. The position of the animals in the behavior testing compartments was randomly assigned.
