# Supplementary material for: Leptin receptor neurons in the dorsomedial hypothalamus input to the circadian feeding network
Source: Sci Adv. 2023 Aug 25;9(34):eadh9570. doi: 10.1126/sciadv.adh9570 (PMC10456850; doi:10.1126/sciadv.adh9570)
Supplement: Supplementary file 1 — Figs. S1 to S13 Legends for table S1 [file sciadv.adh9570_sm.pdf]

Science Advances
AAAS

Supplementary Materials for

Leptin receptor neurons in the dorsomedial hypothalamus input to the circadian feeding network

Qijun Tang *et al.*

Corresponding author: Ali D. Güler, aguler@virginia.edu; John N. Campbell, jnc4e@virginia.edu; Brandon Podyma, podyma@virginia.edu

Sci. Adv. **9**, eadh9570 (2023)
DOI: 10.1126/sciadv.adh9570

The PDF file includes:

Figs. S1 to S13
Legend for table S1

Other Supplementary Material for this manuscript includes the following:

Table S1

SUPPLEMENTAL MATERIALS

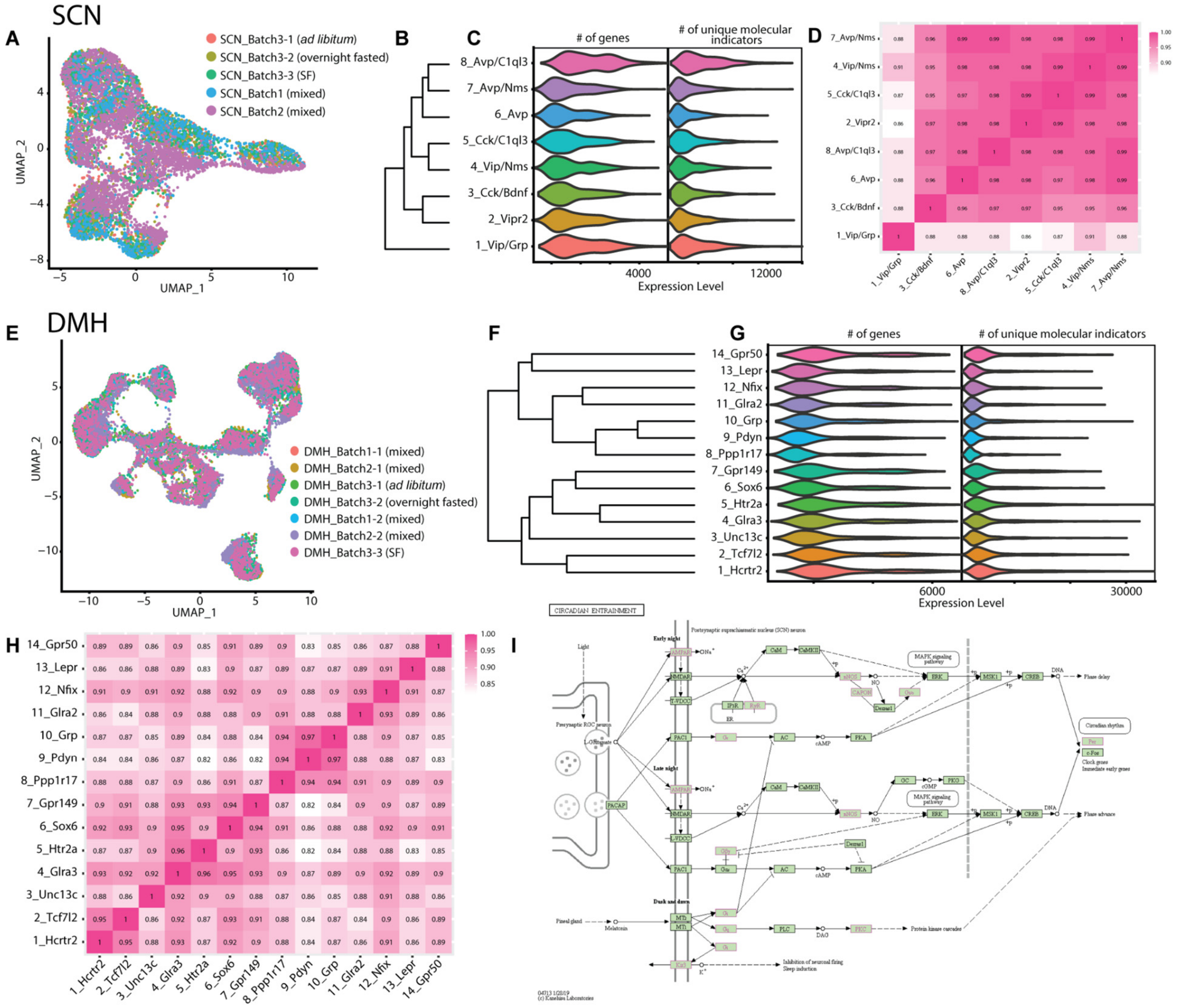

Figure S1. SCN and DMH single nuclei RNA-seq quality metrics and marker expression

- **A.** UMAP of SCN neurons, colored by sequencing library identity, after batch correction.
- **B.** Phylogenetic tree indicating the relatedness of 8 SCN neuronal clusters.
- **C.** Expression level distribution of the number of genes per cluster (left) and number of UMIs (unique molecular identifiers, which represent unique gene transcripts; right) among SCN clusters.
- **D.** Correlation matrix of average expression of all genes between SCN neuronal clusters. Values within the boxes are Pearson correlation coefficients.
- **E.** UMAP of DMH neurons, colored by sequencing library identity, after batch correction.
- **F.** Phylogenetic tree indicating the relatedness of 14 DMH neuronal clusters.
- **G.** Expression level distribution of the number of genes per cluster (left) and number of UMIs (right) among DMH clusters.
- **H.** Correlation matrix of average expression of all genes within DMH neuronal clusters. Values within the boxes are Pearson correlation coefficients.
- **I.** KEGG circadian entrainment pathway map. The genes upregulated (Fig. S4C) in DMH^{LepR} neurons during SF are labeled as pink on the map.

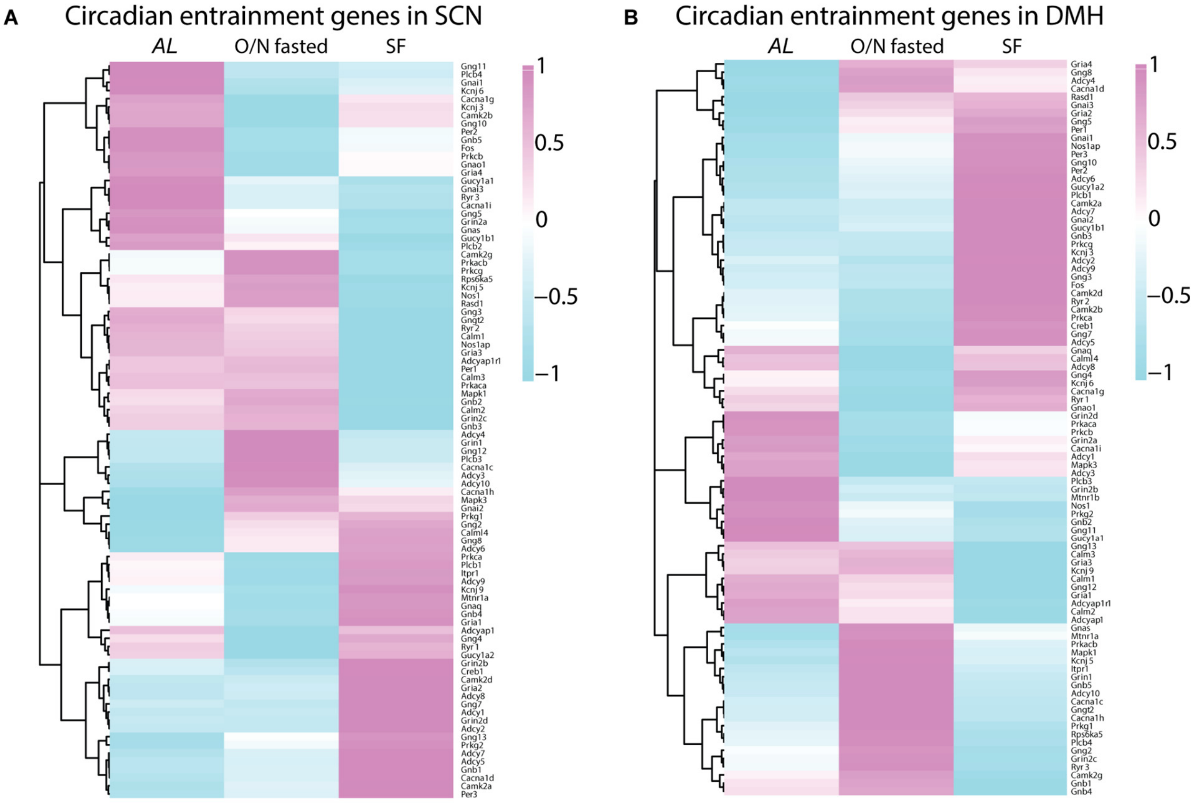

Figure S2. Relative expression level of KEGG circadian entrainment pathway across feeding conditions in SCN and DMH

A. Heatmap of all the genes in the KEGG circadian entrainment pathway across feeding conditions in SCN.

B. Heatmap of all the genes in the KEGG circadian entrainment pathway across feeding conditions in DMH.

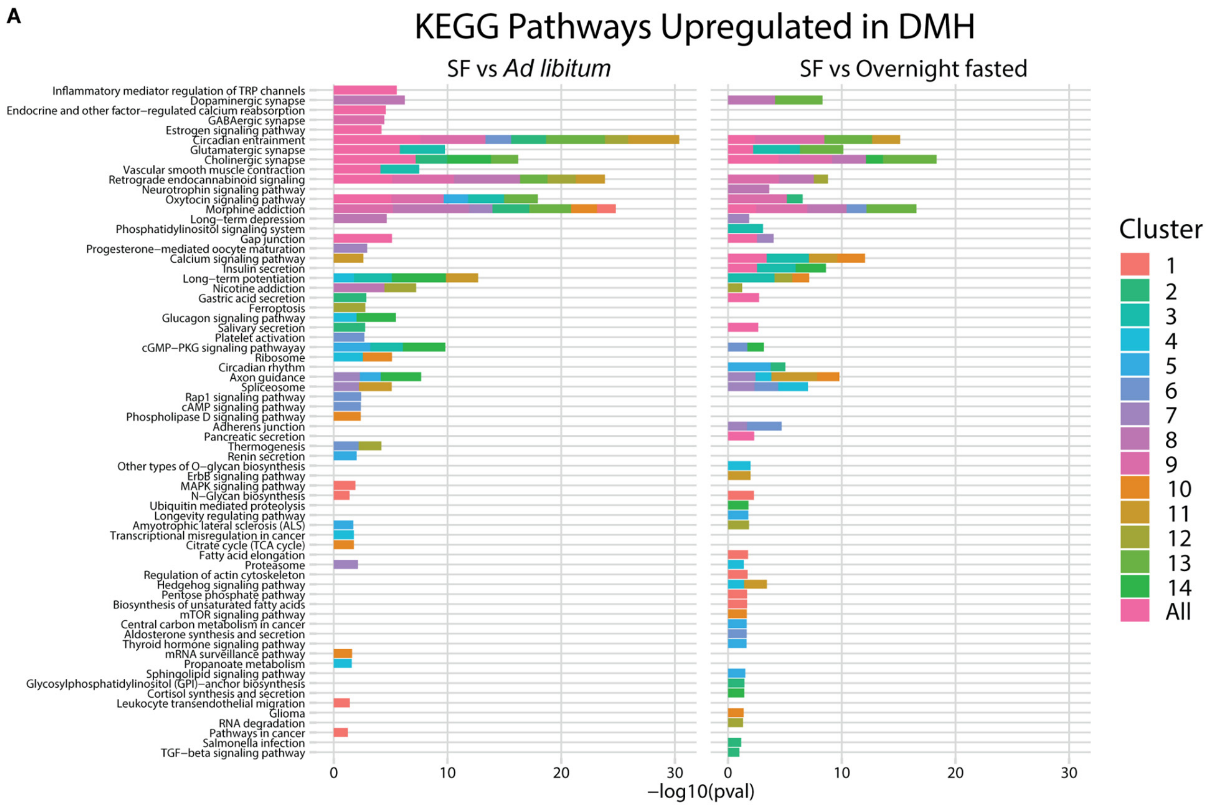

Figure S3. Cluster specific alteration of pathways in the DMH

Gene enrichment analysis comparing pathways upregulated among SF vs *ad libitum* and SF vs fasted feeding conditions in each DMH neuronal cluster and upregulated pathways in all DMH neurons, using KEGG Mouse 2019 database. Bars are colored by clusters. Inclusion criteria required p-value <0.05 and log2 fold change >0.25.

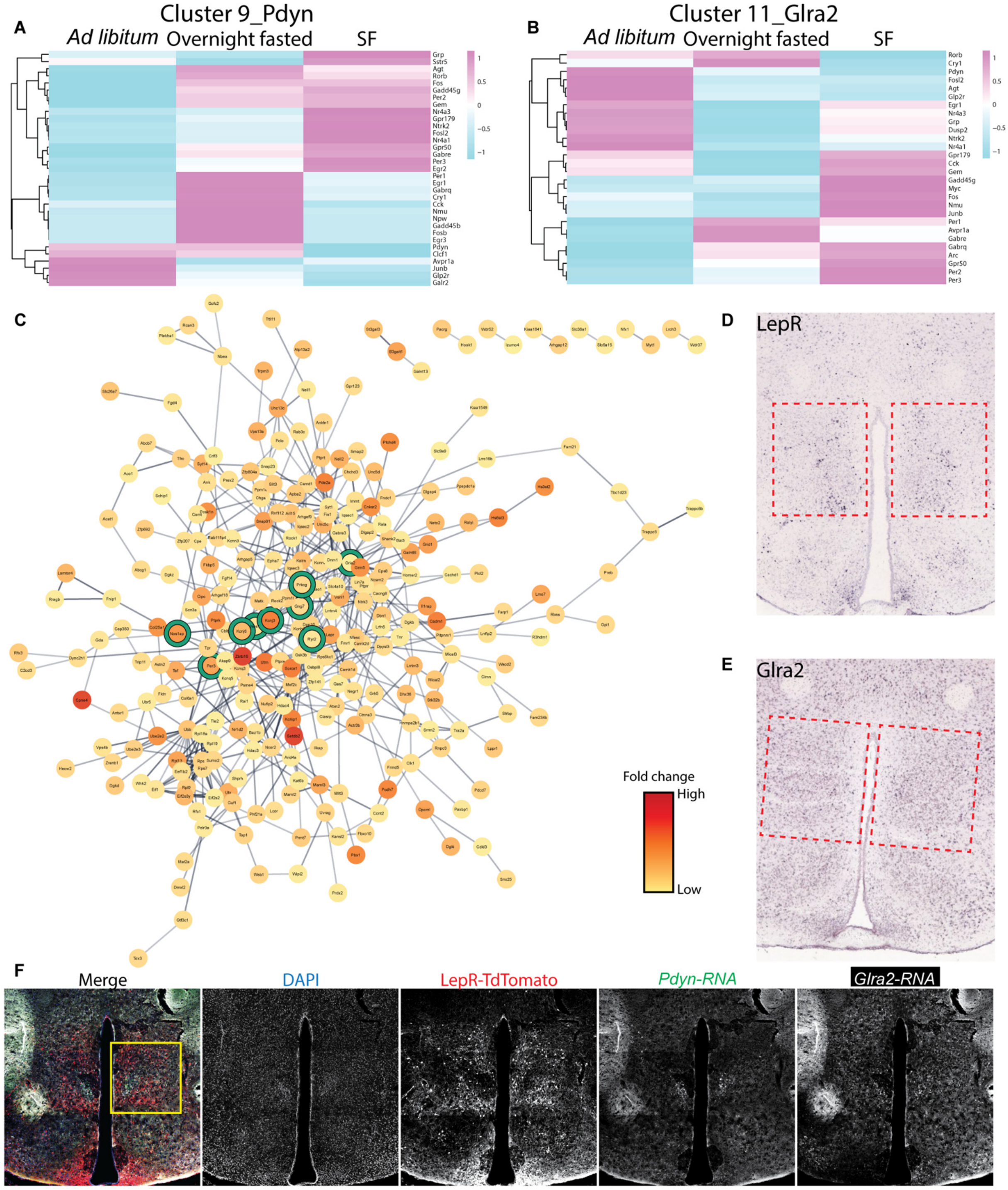

Figure S4. Network analysis of differentially expressed genes in the DMH across feeding conditions

A. Heatmap indicating expression level of genes up and downregulated among feeding conditions in the 9_Pdyn cluster.

B. Heatmap indicating expression level of genes up and downregulated among feeding conditions in the 11_Glra2 cluster.

C. STRING known and predicted protein interactions using Cytoscape platform to visualize molecular networks. Input genes were derived from cluster 13_Lepr differential testing using the Wilcoxon Rank Sum Test to compare SF to *ad libitum* and SF to fasting. Inclusion criteria required False Discovery Rate <0.05 and log2 fold change >0.25. The color of circles indicates the level of average log2 fold change between SF and fasting or *ad libitum* conditions. Functional enrichment of genes involved in circadian entrainment defined by KEGG 2019 database are circled in green.

D-E. *In situ* hybridization (ISH) of the RNA for (D) *Lepr*, and (E) *Glra2* on the brain sections that contain DMH (outlined by red boxes). The images are from Allen Mouse Brain Atlas (https://mouse.brain-map.org/search/index).

F. Representative coronal section image showing the localization of LepR, Pdyn and Glra2 cells in the DMH. LepR cells were marked by LepR-Cre; TdTomato protein, Pdyn and Glra2 cells were marked by RNA FISH. The area indicated by the yellow box was re-imaged and presented as a zoomed-in representative image in Fig. 2H.

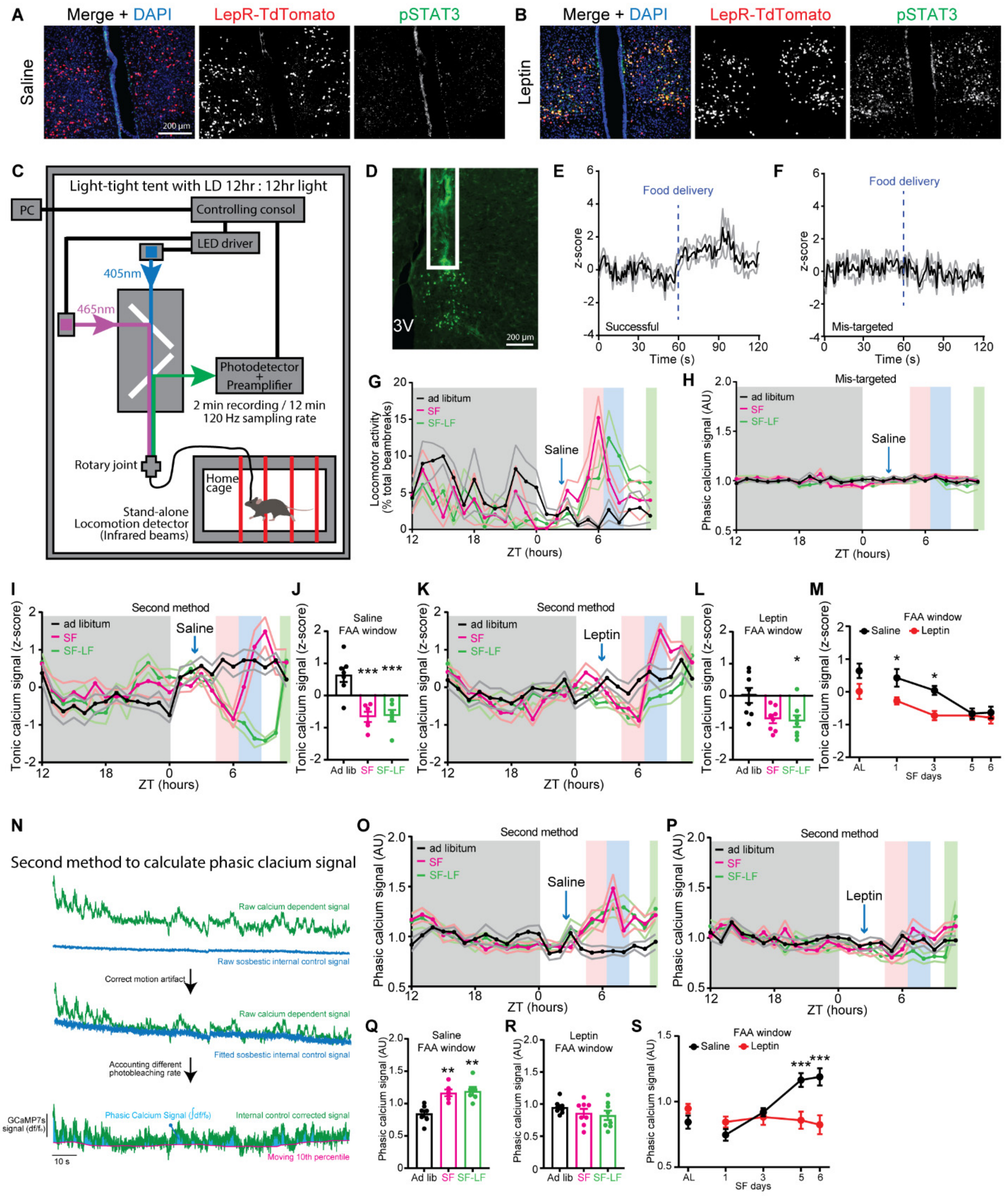

Figure S5. DMH^{LepR} neuron calcium recording during SF

A-B. Representative image showing pSTAT3 expression in the DMH of Lepr-cre; tdTomato mice sacrificed 90 minutes after saline (A) or 5 mg/kg leptin (B) administration.

C. Schematic diagram illustrating long-term fiber photometry recording coupled with locomotor activity monitoring.

D. Representative image showing the expression of GCaMP7s in DMH^{LepR} neurons and fiber optic cannula implant (white box).

E-F. Acute calcium signal response to food delivery on the 5th day of SF, in successful (E) and mis-targeted (F) mice**.** The mis-targeted animals in (D) were excluded for the analysis in Fig. 3-4. n=6 mice/group.

G. Locomotor activity of mis-targeted mice during SF. These mice developed strong FAA. n=5.

H. Average phasic GCaMP7s signal of DMH^{LepR} neurons in the mis-targeted mice at 2 days before SF (black, *ad libitum*), 5th day of saline treated SF (magenta, SF), or 6th day of SF where saline injection was withheld and food delivery was delayed for 3.5 hours (green, SF-LF). n=6. The unaltered phasic calcium signal of these mis-targeted mice during SF suggests that the pre-meal elevation of phasic calcium signal is not due to artifact of increased locomotor activity.

I. Average tonic calcium signal (z-score) of DMH^{LepR} neurons from saline control group 2 days before SF (black, *ad libitum*), 5th day during treatment (magenta, SF), and 6th day where saline injection was withheld and food delivery was delayed for 3.5 hours (green, SF-LF: late feeding).

J. Quantification of the tonic calcium signal from saline treated mice in the FAA window (average of ZT5-6) from (I). Mixed-effects (REML) analysis with Bonferroni post hoc comparison; n = 6-7 / group; F (2, 16) = 15.41, p=0.0002.

K. Average tonic calcium signal (z-score) of DMH^{LepR} neurons from leptin group 2 days before SF (black, *ad libitum*), 5th day during treatment (magenta, SF), and 6th day where leptin injection was withheld and food delivery was delayed for 3.5 hours (green, SF-LF: late feeding).

L. Quantification of the tonic calcium signal from leptin treated mice in the FAA window (average of ZT5-6) from (K). Repeated measures one-way ANOVA with Bonferroni post hoc comparison; n = 8 / group; F (2, 14) = 4.346, p=0.0340.

M. Quantification of the development of the tonic calcium signal (z-score) during FAA. AL indicates *ad libitum* condition two days prior to initiation of drug administration. Mixed-effects (REML) analysis with Bonferroni post hoc comparison; n = 6-8 / group; $F_{treatment}$ (1, 63) = 16.08, p=0.0002.

N. Example data trace illustrating second method of calculating phasic calcium signal. See also the Material and Methods section for more details.

O. Average phasic calcium signal (second method) of DMH^{LepR} neurons from saline control group 2 days before SF (black, *ad libitum*), 5th day during treatment (magenta, SF), and 6th day where

saline injection was withheld and food delivery was delayed for 3.5 hours (green, SF-LF: late feeding). Normalized to average dark phase signal (ZT12-ZT0).

P. Average phasic calcium signal (second method) of DMH^{LepR} neurons from leptin group 2 days before SF (black, *ad libitum*), 5th day during treatment (magenta, SF), and 6th day where leptin injection was withheld and food delivery was delayed for 3.5 hours (green, SF-LF: late feeding). Normalized to average dark phase signal (ZT12-ZT0).

Q. Quantification of the phasic calcium signal from saline treated mice in the FAA window (average of ZT5-6) from (O). Mixed-effects (REML) analysis with Bonferroni post hoc comparison; n = 6-7 / group; F (2, 10) = 14.82, $p=0.0010$.

R. Quantification of the phasic calcium signal from leptin treated mice in the FAA window (average of ZT5-6) from (P). Repeated measures one-way ANOVA with Bonferroni post hoc comparison; n = 8 / group; F (2, 14) = 1.986, $p=0.1741$.

S. Quantification of the development of the phasic calcium signal during FAA. AL indicates *ad libitum* condition two days prior to initiation of drug administration. Mixed-effects (REML) analysis with Bonferroni post hoc comparison; n = 6-8 / group; $F_{treatment * time}$ (4, 50) = 12.52, $p<0.0001$.

Data are represented as mean ± SEM. *$p < 0.05$; **$p < 0.01$; ***$p < 0.001$; ns, not significant.

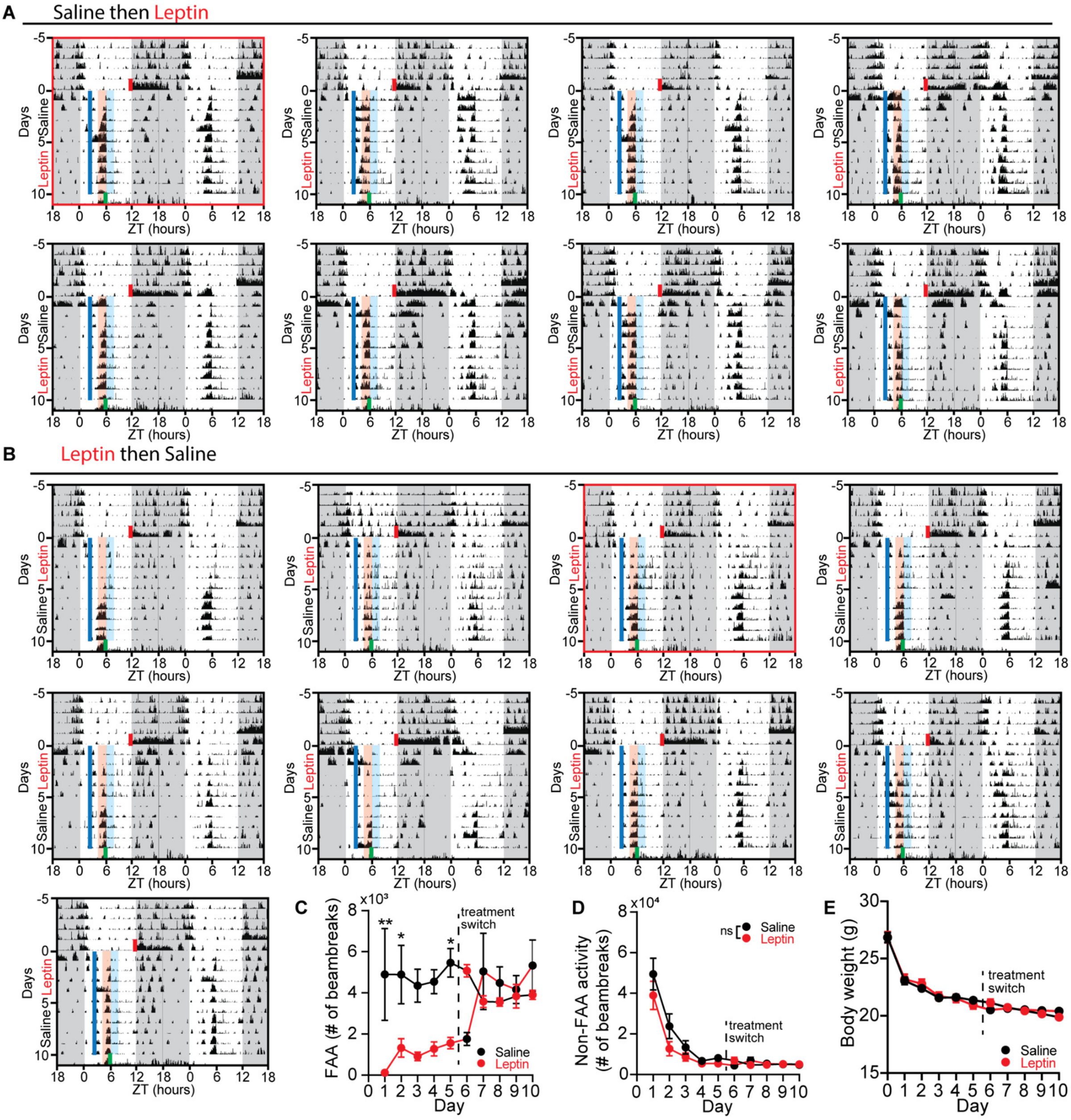

Figure S6. Pre-FAA leptin suppresses the development but not maintenance of FAA

A-B. Actograms of all animals that received (A) saline then leptin or (B) leptin then saline, during scheduled feeding (SF). The actograms of animals that have minimum sum of the square of the residuals to the average FAA value were used as representative figures in Fig. 5, and depicted with red boxes.

C. Absolute locomotor activity during the FAA window, 2 hours prior to food delivery time. Repeated measures two-way ANOVA with Bonferroni post hoc comparison; n = 8-9 / group; $F_{treatment}$ (1, 15) = 22.3, $p<0.001$.

D. Absolute locomotor activity for the 22 hours per day outside of the FAA window. Repeated measures two-way ANOVA; n = 8-9 / group; $F_{treatment}$ (1, 15) = 1.639, p=0.2198.

E. Body weight of animals during SF. Repeated measures two-way ANOVA; n = 8-9 / group; $F_{treatment}$ (1, 15) = 0.007550, p=0.9319; $F_{treatment*time}$ (10, 150) = 2.633, p=0.0056.

Data are represented as mean ± SEM. *p < 0.05; **p < 0.01; ***p < 0.001; ns, not significant.

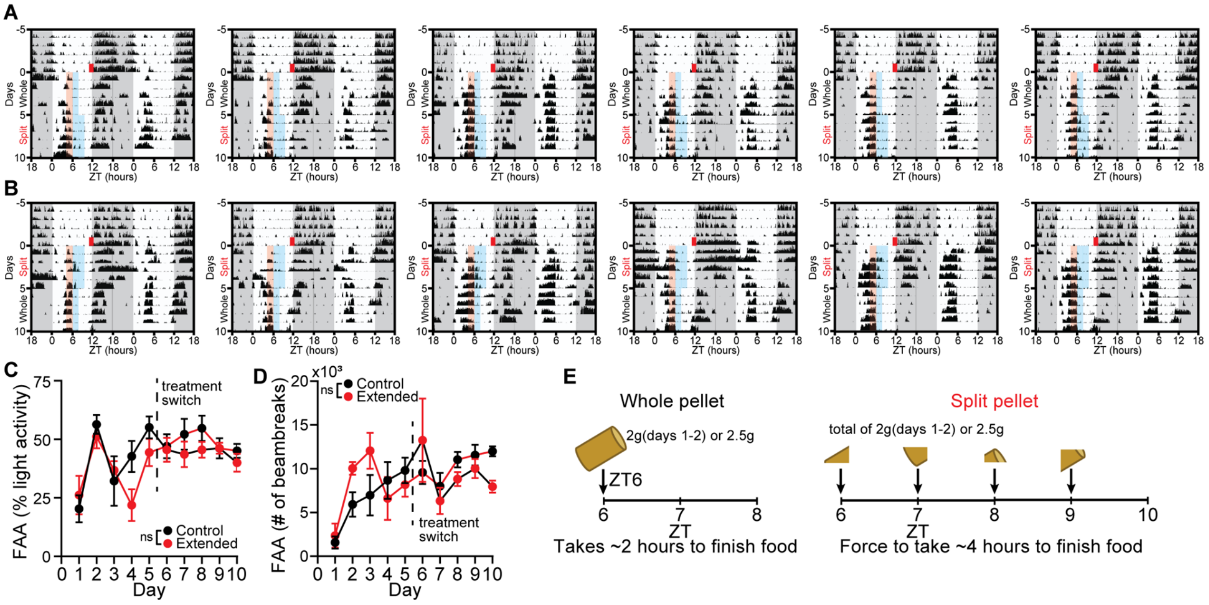

Figure S7. Extended duration of food intake does not confound the development of FAA

A-B. Actograms of all animals that received (A) control (whole pellet) then extended (same size pellet split up into 4 pellets) or (B) extended then control SF paradigm. Briefly, mice in the extended SF paradigm received one quarter of a pellet of food every hour for 4 hours, while mice in the control SF paradigm received one whole pellet at the first hour (see Methods and (E) for details).

C. Quantification of FAA in extended SF experiment. Illustrated as a percentage of light-phase activity, without excluding any light-phase activity. Repeated measures two-way ANOVA with Bonferroni post hoc comparison; n = 6 / group; $F_{treatment}$ (1, 10) = 0.004799, p=0.9461.

D. Absolute locomotor activity during the FAA window in extended SF experiment. FAA window is defined as 2 hours prior to food delivery time. Repeated measures two-way ANOVA with Bonferroni post hoc comparison; n = 6 / group; $F_{treatment}$ (1, 10) = 1.362, p=0.2703.

E. Diagram illustrating the comparison between whole pellet and split pallet groups.

Data are represented as mean ± SEM. *p < 0.05; **p < 0.01; ***p < 0.001; ns, not significant.

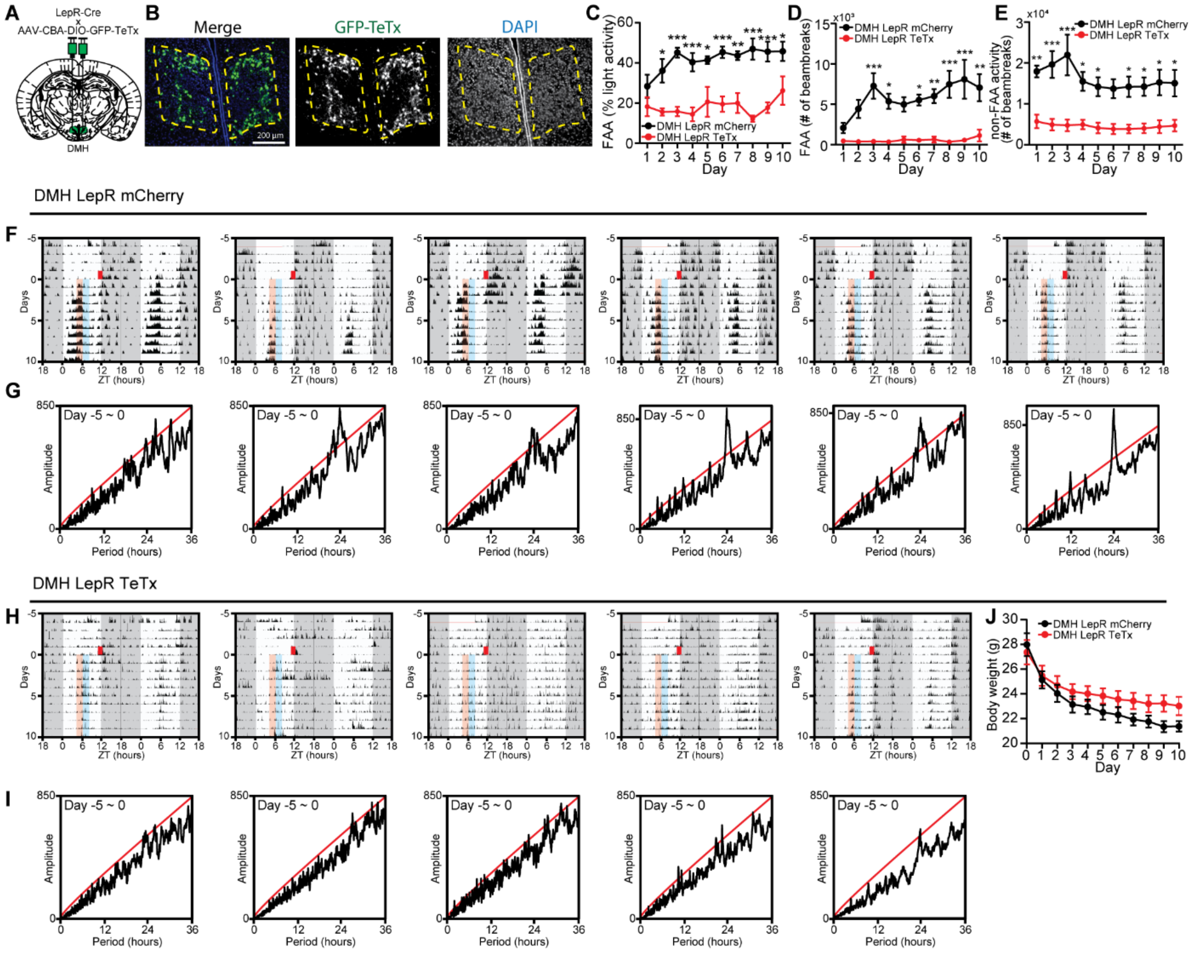

Figure S8. Silencing of DMH^{LepR} neurons impairs general behavioral rhythmicity and FAA

A. Schematic diagram illustrating DMH^{LepR} neuron silencing by bilateral injection of AAV-CBA-DIO-GFP-TeTx to the DMH of LepR Cre mice.

B. Representative images showing the expression of GFP-TeTx in DMH^{LepR} neurons.

C. Quantification of FAA. FAA is defined as the locomotor activity in the two-hour window prior to food delivery as a percentage of light-phase activity. Repeated measures two-way ANOVA with Bonferroni post hoc comparison; n = 5-6 / group; F_{virus} (1, 9) = 65.57, p<0.0001.

D. Absolute locomotor activity during the FAA window on SF. Repeated measures two-way ANOVA with Bonferroni post hoc comparison; n = 5-6 / group; F_{virus} (1, 9) = 58.66, p<0.0001.

E. Absolute locomotor activity for the 22 hours per day other than FAA. Repeated measures two-way ANOVA; n = 5-6 / group; F_{virus} (1, 9) = 23.13, p=0.0010.

F-I. Actograms (F, H) and periodogram (G, I) of all DMH^{LepR} mCherry (F-G) and DMH^{LepR} TeTx (H-I) mice on SF. The Chi-square significance line of p=0.001 is depicted as a red line in the periodograms (G, I).

J. Body weight of animals during SF. Repeated measures two-way ANOVA; n = 5-6 / group; F_{virus} (1, 9) = 1.330, p=0.2785; $F_{virus*time}$ (10, 90) = 5.752, p<0.0001.

Data are represented as mean ± SEM. *p < 0.05; **p < 0.01; ***p < 0.001; ns, not significant.

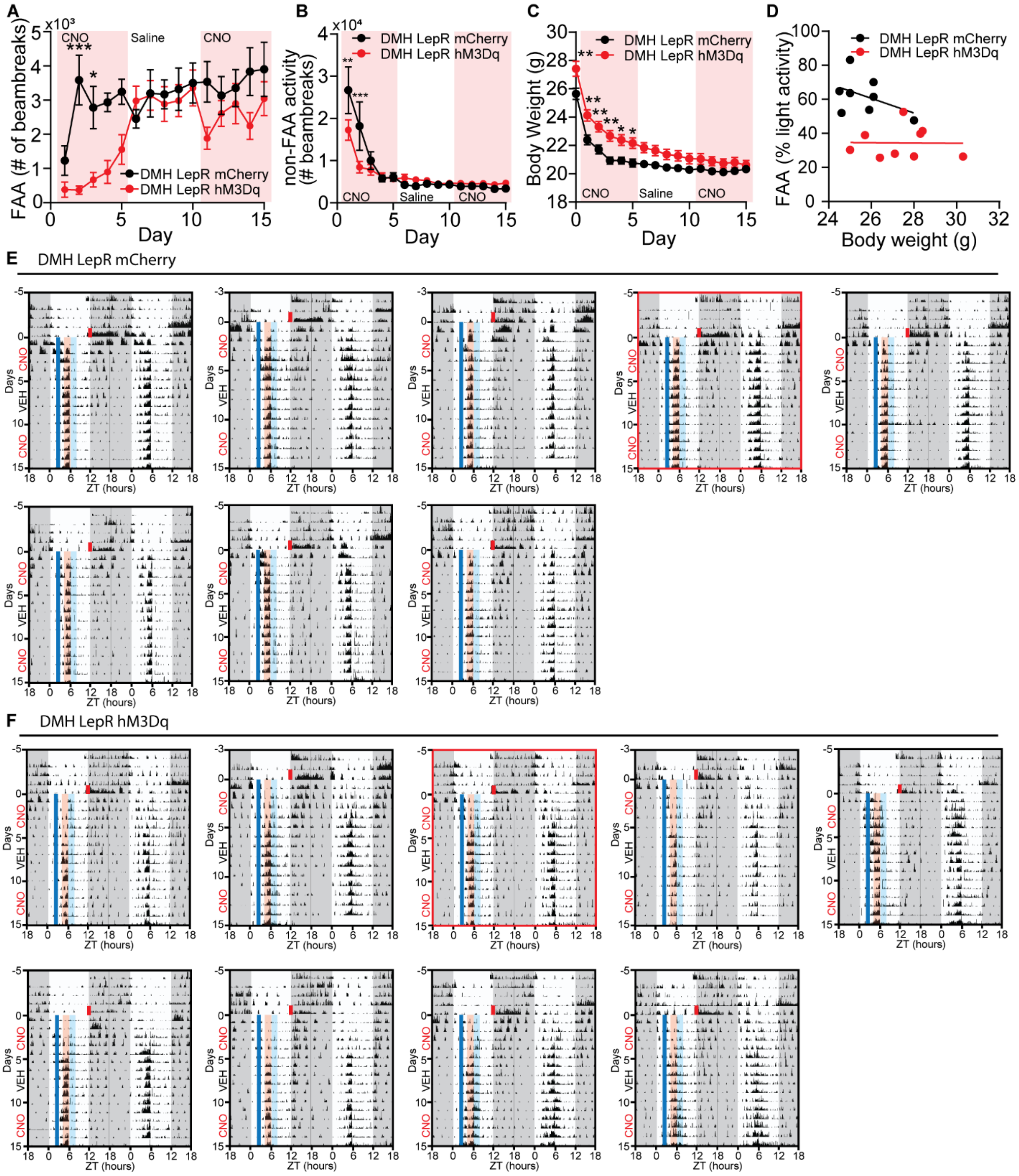

Figure S9. Pre-FAA activation of DMH^{LepR} neurons suppresses development of FAA

A. Absolute locomotor activity during the FAA window on SF. Repeated measures two-way ANOVA with Bonferroni post hoc comparison; n = 8-9 / group; $F_{time*virus}$ (14, 210) = 4.333, p<0.001.

B. Absolute locomotor activity for the 22 hours per day outside of the FAA window. Repeated measures two-way ANOVA with Bonferroni post hoc comparison; n = 8-9 / group; $F_{time*virus}$ (14, 210) = 3.491, p<0.001.

C. Body weight during SF. Repeated measures two-way ANOVA with Bonferroni post hoc comparison; n = 8-9 / group; F_{virus} (1, 15) = 6.533, p=0.0219.

D. Correlation between initial body weight (day before SF) with FAA level on 5th day of SF. Higher starting body weight of hM3Dq expressing animals was not the cause of dampened development of FAA.

E-F. Actograms of all DMH^{LepR} mCherry (E) and DMH^{LepR} hM3Dq (F) mice on SF. The actograms of animals that have minimum sum of the square of the residuals to the average FAA value were used as representative figures in Fig. 6, and depicted with red boxes.

Data are represented as mean ± SEM. *p < 0.05; **p < 0.01; ***p < 0.001; ns, not significant.

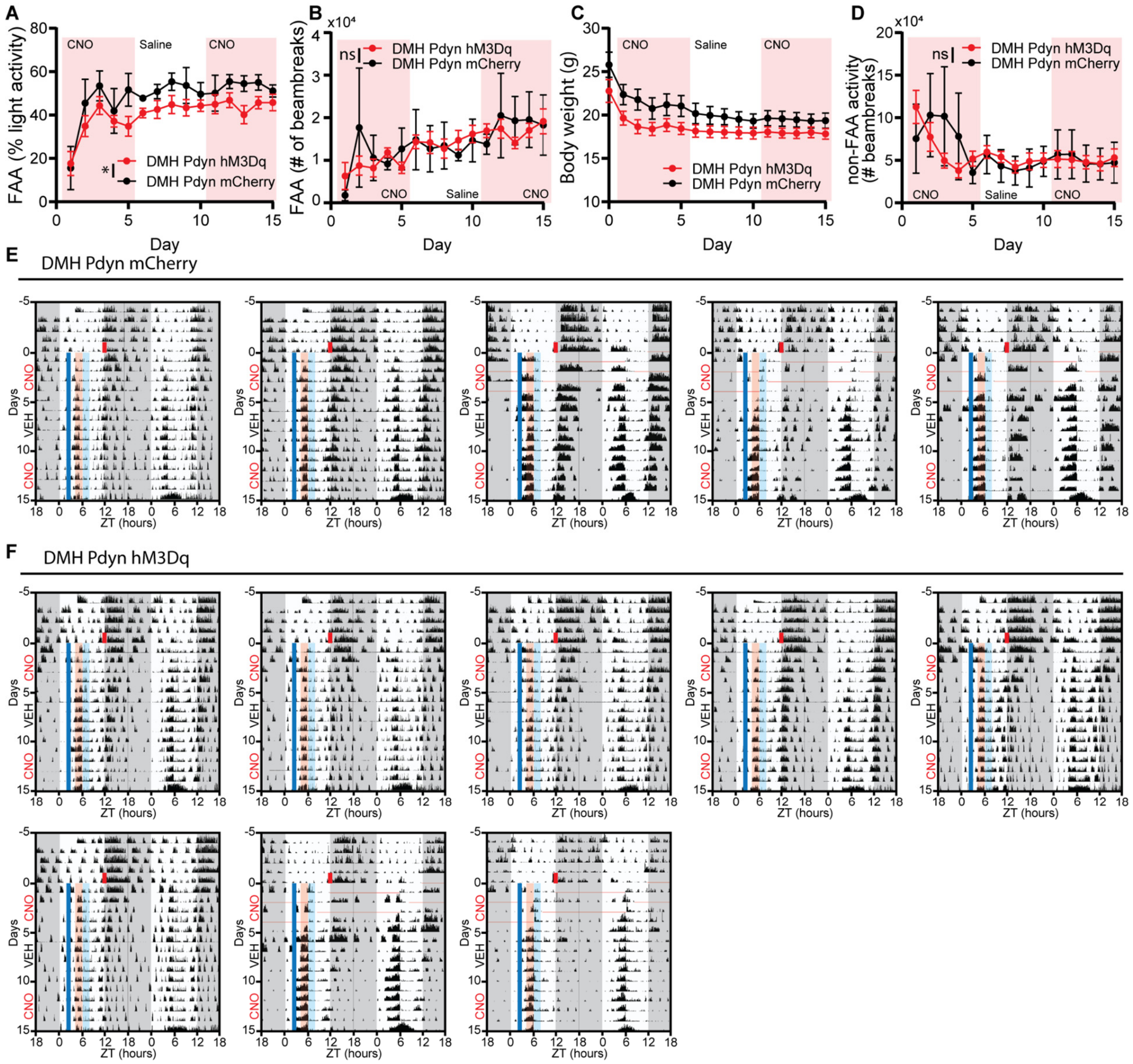

Figure S10. Chemogenetic activation of DMH^{Pdyn} neurons slightly inhibits the robustness of FAA

A. Quantification of FAA from DMH^{Pdyn} mCherry and DMH^{Pdyn} hM3Dq mice on SF that received 0.3mg/kg CNO (SF days 1-5), saline (SF days 6-10), and 0.3 mg/kg CNO (SF days 11-15) injection at ZT2.5 during SF. Pink shading indicates days with CNO injection. No shading indicates saline injection. Mixed-effects (REML) analysis with Bonferroni post hoc comparison; n = 5-8 / group; F_{virus} (1, 11) = 5.601, p=0.0374.

B. Absolute locomotor activity during the FAA window on SF. Mixed-effects (REML) analysis; n=5-8 / group; F_{virus} (1, 11) = 0.001101, p=0.9741; $F_{virus*time}$ (14, 144) = 1.106, p=0.3576.

C. Body weight during SF. Repeated measures two-way ANOVA with Bonferroni post hoc comparison; n = 5-8 / group; F_{virus} (1, 11) = 2.827, p=0.1208; $F_{time*virus}$ (15, 165) = 2.834, p=0.0006.
D. Absolute locomotor activity for the 22 hours per day outside of the FAA window. Mixed-effects (REML) analysis; n=5-8 / group; F_{virus} (1, 11) = 0.03343, p=0.8582; $F_{virus\ *time}$ (14, 145) = 1.601, p=0.0853.
E-F. Actograms of all DMH^{Pdyn} mCherry (E) and DMH^{Pdyn} hM3Dq (F) mice on SF. Horizontal red lines indicate the missing data due to technical failure.
Data are represented as mean ± SEM. *p < 0.05; **p < 0.01; ***p < 0.001; ns, not significant.

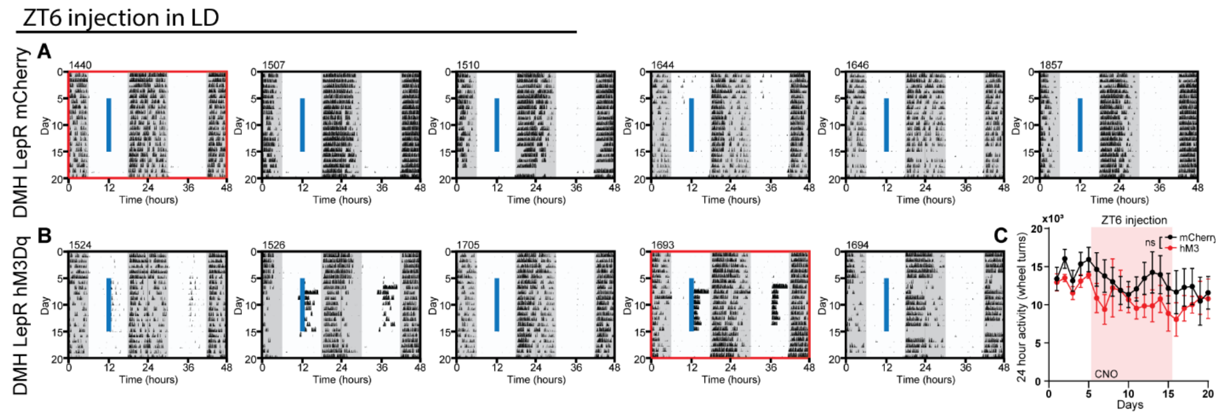

Figure S11. Repetitive activation of DMH^{LepR} neurons alters circadian locomotor activity under *ad libitum* feeding conditions in LD

A-B. Actograms of all LepR Cre animals bilaterally injected with (A) AAV-hSyn-DIO-mCherry or (B) AAV-hSyn-DIO-hM3Dq-mCherry and injected with CNO at ZT6 (solid blue line) in 12-12 LD. The actograms used as representative figures in Fig. 7 are depicted with red boxes.

C. 24-hour total wheel revolutions of DMH^{LepR} mCherry and DMH^{LepR} hM3Dq animals before, during, and after CNO injections at ZT6 in 12-12 LD. Pink shading represents days with CNO injection. Repeated measures two-way ANOVA; n = 5-6 / group; F_{virus} (1, 9) = 0.7855, p=0.3985; $F_{virus*time}$ (19, 171) = 0.9782, p=0.4890.

Data are represented as mean ± SEM. *p < 0.05; **p < 0.01; ***p < 0.001; ns, not significant.

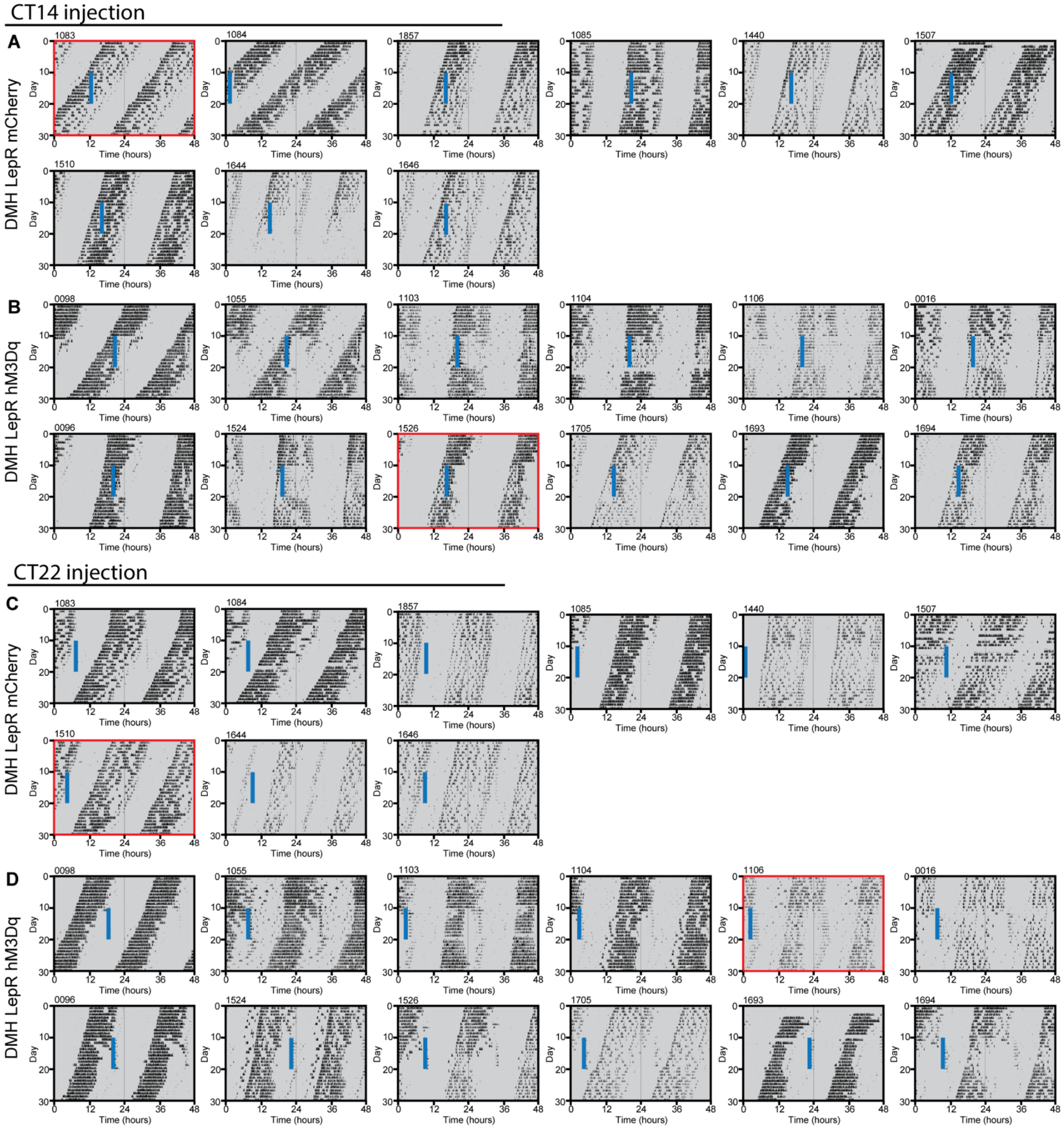

Figure S12. Repetitive activation of DMH^{LepR} neurons alters circadian locomotor activity under *ad libitum* feeding conditions in DD

A-B. Actograms of all LepR Cre animals bilaterally injected with (A) AAV-hSyn-DIO-mCherry or (B) AAV-hSyn-DIO-hM3Dq-mCherry and injected with CNO at ~CT14 (solid blue line). The actograms used as representative figures in Fig. 8 are depicted with red boxes.

C-D. Actograms of all LepR Cre animals bilaterally injected with (C) AAV-hSyn-DIO-mCherry or (D) AAV-hSyn-DIO-hM3Dq-mCherry and injected with CNO at ~CT22 (solid blue line). The actograms used as representative figures in Fig. 8 are depicted with red boxes. Notably, animal

#1084 in (C) was excluded from quantification in Fig. 8H-I, because the injection time on the 10th day was near the locomotor activity onset (<3 hours) of the following day, due to the short free-running period.

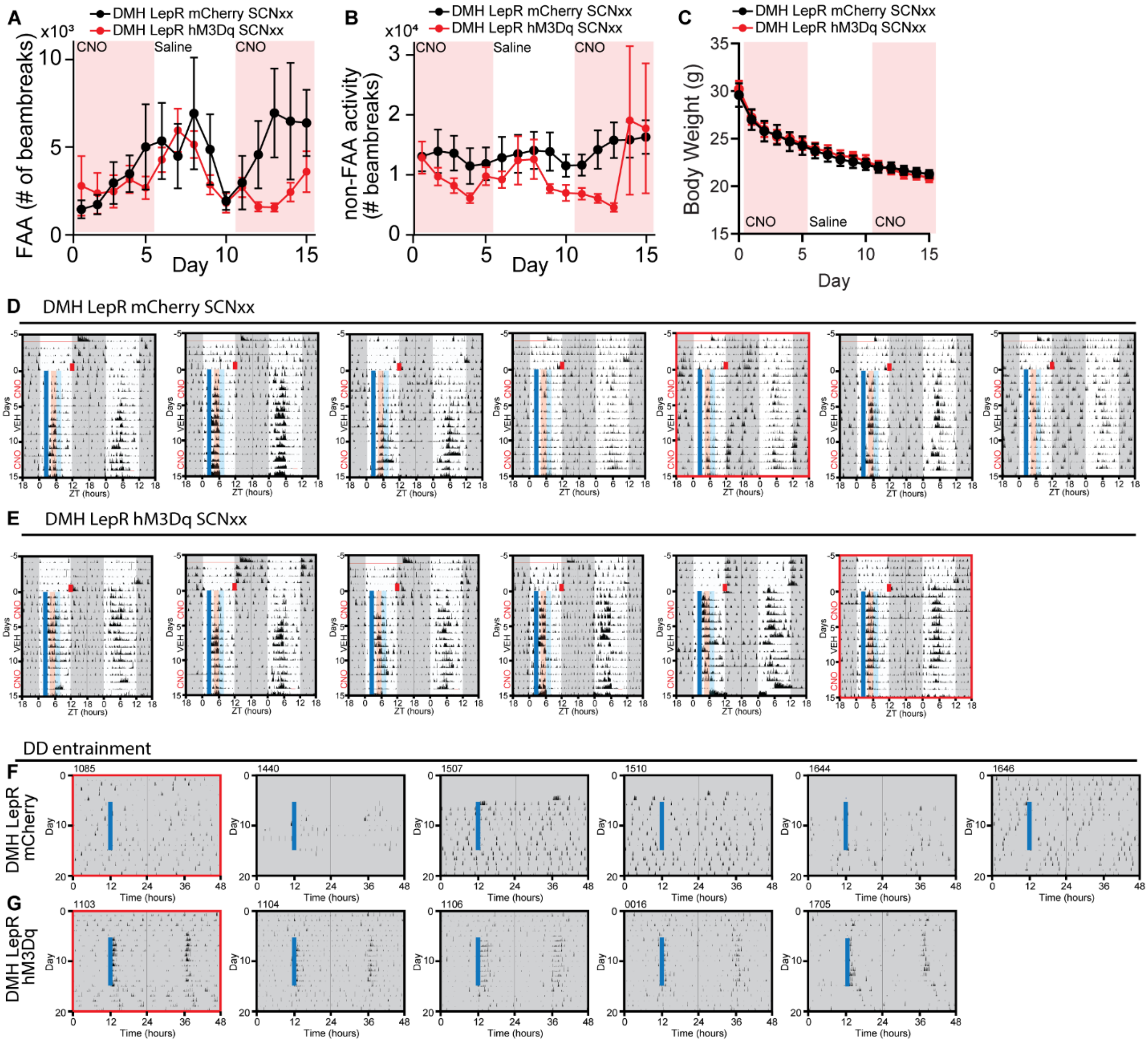

Figure S13. DMH^{LepR} neuron activation fails to suppress the FAA development in SCN lesioned mice

A. Absolute locomotor activity of SCN lesioned animals during the FAA window on SF. Repeated measures two-way ANOVA; n = 6-7 / group; F_{virus} (1, 11) = 0.6742, p=0.4290.

B. Absolute locomotor activity of SCN lesioned animals for the 22 hours per day outside of the FAA window. Repeated measures two-way ANOVA; n = 6-7 / group; F_{virus} (1, 11) = 1.004, p=0.3378.

C. Body weight of SCN lesioned animals during SF. Repeated measures two-way ANOVA; n = 7 / group; F_{virus} (1, 12) = 0.01935, p=0.8917.

D-E. Actograms of all SCN lesioned DMH^{LepR} mCherry (D) and DMH^{LepR} hM3Dq (E) mice on SF. The actograms of animals that have minimum sum of the square of the residuals to the average FAA value were used as representative figures in Fig. 10 and depicted with red boxes.

F-G. Actograms of all LepR Cre animals with electrolytic lesions of the SCN and bilaterally injected with (F) AAV-hSyn-DIO-mCherry or (G) AAV-hSyn-DIO-hM3Dq-mCherry and injected with CNO every 24 hours for 10 days (solid blue line). The actograms used as representative figures in Fig. 10 are depicted with red boxes.

Data are represented as mean ± SEM. $*p < 0.05$; $**p < 0.01$; $***p < 0.001$; ns, not significant.

Table S1. Individual data value of bioluminescence experiments
